# Gut microbiota-derived metabolites mediate the neuroprotective effect of melatonin in cognitive impairment induced by sleep deprivation

**DOI:** 10.1186/s40168-022-01452-3

**Published:** 2023-01-31

**Authors:** Xintong Wang, Zixu Wang, Jing Cao, Yulan Dong, Yaoxing Chen

**Affiliations:** 1 Neurobiology Laboratory, College of Veterinary Medicine, China Agricultural University, Haidian, Beijing, 100193 China; 2 Department of Nutrition and Health, China Agricultural University, Haidian, Beijing, 100193 China

**Keywords:** Sleep deprivation, Hippocampus, Microbial–gut–brain axis, Cognitive impairment, Melatonin

## Abstract

**Background:**

Sleep loss is a serious global health concern. Consequences include memory deficits and gastrointestinal dysfunction. Our previous research showed that melatonin can effectively improve cognitive impairment and intestinal microbiota disturbances caused by sleep deprivation (SD). The present study further explored the mechanism by which exogenous melatonin prevents SD-induced cognitive impairments. Here, we established fecal microbiota transplantation, *Aeromonas* colonization and LPS or butyrate supplementation tests to evaluate the role of the intestinal microbiota and its metabolites in melatonin in alleviating SD-induced memory impairment.

**Results:**

Transplantation of the SD-gut microbiota into normal mice induced microglia overactivation and neuronal apoptosis in the hippocampus, cognitive decline, and colonic microbiota disorder, manifesting as increased levels of *Aeromonas* and LPS and decreased levels of *Lachnospiraceae_NK4A136* and butyrate. All these events were reversed with the transplantation of SD + melatonin-gut microbiota. Colonization with *Aeromonas* and the addition of LPS produced an inflammatory response in the hippocampus and spatial memory impairment in mice. These changes were reversed by supplementation with melatonin, accompanied by decreased levels of *Aeromonas* and LPS. Butyrate administration to sleep-deprived mice restored inflammatory responses and memory impairment. In vitro, LPS supplementation caused an inflammatory response in BV2 cells, which was improved by butyrate supplementation. This ameliorative effect of butyrate was blocked by pretreatment with MCT1 inhibitor and HDAC3 agonist but was mimicked by TLR4 and p-P65 antagonists.

**Conclusions:**

Gut microbes and their metabolites mediate the ameliorative effects of melatonin on SD-induced cognitive impairment. A feasible mechanism is that melatonin downregulates the levels of *Aeromonas* and constituent LPS and upregulates the levels of *Lachnospiraceae_NK4A136* and butyrate in the colon. These changes lessen the inflammatory response and neuronal apoptosis in the hippocampus through crosstalk between the TLR4/NF-κB and MCT1/ HDAC3 signaling pathways.

Video Abstract

**Supplementary Information:**

The online version contains supplementary material available at 10.1186/s40168-022-01452-3.

## Background

Sleep deprivation (SD) disrupts millions of people’s lives worldwide and profoundly impacts cognition and physical performance [1, 2]. Individuals with acute SD have increased risks of Alzheimer’s disease and cardiovascular disease, as well as an increased level of systemic inflammation [3–5]. SD affects the whole body and involves systematic damage to multiple tissues. However, the underlying mechanisms contributing to cognitive impairment caused by SD remain unclear.

The gut microflora, also known as the second brain, may influence brain homeostasis through the microbial–gut–brain axis under both physiological and pathological conditions [6]. In healthy individuals, the stable gut microbiota composition plays a critical role in sustaining the balance between intestinal barrier integrity and inflammation, thus positively regulating brain function through the microbiota–gut–brain axis [7–9]. However, many pathological changes in intestinal microflora have been reported in patients with insomnia [10]. We previously reported disturbed intestinal microflora and intestinal barrier dysfunction in sleep-deprived mice [11]. Moreover, healthy mice that received gut microbiota transplantation from donors with insomnia showed cognitive dysfunction [12]. Collectively, the findings suggest that gut microbiota dysbiosis plays a pivotal role in cognitive impairment caused by SD. However, the underlying mechanism requires further investigation.

Dysbiosis of intestinal microflora can lead to intestinal inflammation and neuroinflammation [13, 14]. The increase in opportunistic pathogens is one of the possible consequences of dysbiosis of the intestinal microbiota, which can destabilize intestinal tight junction proteins, leading to disruption of the intestinal integrity barrier and an increase in permeability, called leaky gut [15, 16]. *Aeromonas*, an opportunistic pathogen, can induce intestinal inflammation and cause extraintestinal inflammatory responses [17, 18]. Breakage of the intestinal barrier can lead to the entry of large numbers of microorganisms or microbial constituents, such as lipopolysaccharide (LPS), into circulation, thus causing systemic inflammation [19]. The permeability of the blood–brain barrier (BBB) is also threatened by high levels of pro-inflammatory molecules in the systemic circulation, resulting in its blocking effect on LPS and inflammatory cytokines being diminished [20]. LPS that enters the brain can bind to Toll-like receptor 4 (TLR4) receptors on microglia, leading to the synthesis and secretion of large amounts of pro-inflammatory cytokines [21]. Hippocampal neurons are also damaged in an inflammatory microenvironment [22]. However, targeting the gut–brain axis to alleviate cognitive impairment in sleep-deprived mice remains unexplored.

Melatonin (N-acetyl-5-methoxytryptamine, Mel) is the main hormone secreted by the pineal gland. Mel is an indoleamine with antioxidant, chronobiotic, and anti-inflammatory properties [23]. Mel can reduce organ inflammation and reshape intestinal microflora in animals and humans [24, 25]. In addition, Mel attenuates inflammatory osteolysis induced by titanium nanoparticles by enriching microflora that produces short-chain fatty acids (SCFAs) and elevating the metabolite butyrate to activate the GPR109A receptor [26]. SCFAs are primarily produced in the large intestine through anaerobic bacterial fermentation. They maintain intestinal immune function and regulate gut barrier function [27]. SCFAs can enter the circulatory system and may signal the brain [28]. SCFAs also regulate the maturation and function of microglia and prevent neuroinflammatory processes [29, 30]. Although we previously found that Mel alleviates cognitive impairment in sleep-deprived mice, its mechanism of action remains unclear.

Our previous studies showed that Mel can effectively relieve acute SD-induced cognitive impairment [2] and gut microbiota disorders [11]. The present study further explored the effect of the gut microbiota and its metabolites on the improvement of SD-induced cognitive impairment by Mel. The present study had three facets. The first was to verify the core role of the disorder of the gut microbiota and its metabolite in SD-induced cognitive impairment using fecal microbiota transplantation (FMT). Secondly, colonization of *Aeromonas veronii* (*A. veronii*) and addition of LPS to control mice, or supplementing butyrate to SD mice confirm Mel improve the cognitive impairment induced by SD. Thirdly, the signaling pathway in Mel-mediated butyrate relief of LPS-induced inflammatory response was explored in vitro.

## Methods

### Animals and experimental design

All animal experiments in this study were approved by the Animal Welfare Committee of the Agricultural Research Organization, China Agricultural University (approval no. CAU201709112). Male ICR mice (8 weeks old; 35–40 g) were purchased from the Beijing Vital River Laboratory (Beijing, China). The mice were placed in cages and maintained under standard environmental conditions of temperature (21 ± 1 °C) and relative humidity (50 ± 10%), with a regular 14-h light/10-h dark cycle. The light was turned on at 7:00 h and turned off at 21:00 h. All mice were acclimated for 1 week before the experiments.

### FMT experiment

**Fig. 1:**
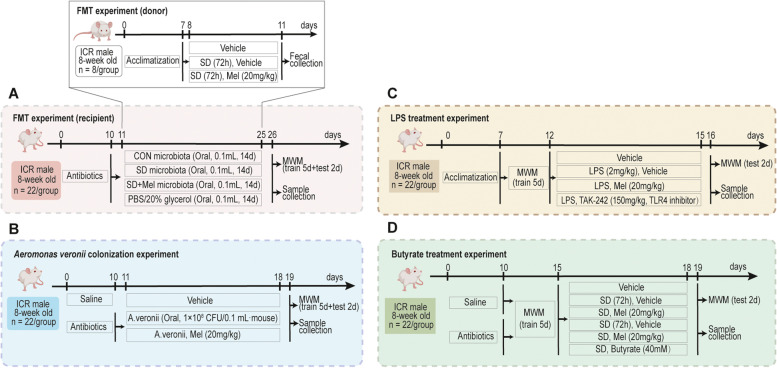
Schematic and timeline of the experimental model. **A** Fecal microbiota transplantation (FMT) experiment. **B** *Aeromonas veronii* colonization experiment. **C** LPS treatment experiment. **D** Butyrate treatment experiment. Mel: melatonin, MWM: Morris water maze, Vehicle: 2% ethanol sterile saline. SD: sleep deprivation, TAK-242: TLR4. inhibitor

To investigate the effects of gut microbiota on cognitive impairment induced by SD, mice were divided into donor and recipient groups (Fig. 1A). Donor mice were further divided into the control (CON), SD, and SD supplemented with Mel (20 mg/kg) (SD + Mel) groups. Acute SD of the mice began at 8 am every day for 3 days using a modified multiple-platform water bath, as described previously [11]. Mel (20 mg/kg; approximately 8 mg, M5250; Sigma-Aldrich, St. Louis, MO, USA) was dissolved in 20 µL of ethanol, followed by the addition of 1 mL of sterile saline to prepare a solution used for intraperitoneal injection (final ethanol concentration: 2%). The control mice received an equivalent amount of vehicle (2% ethanol in sterile saline). SD mice were administered intraperitoneal injection lacking Mel (vehicle only, SD group) and containing 20 mg/kg Mel (SD + Mel group). The injection was delivered 60 min before SD, followed by one injection each day at 7:00 h for 3 days. Before FMT, for substantial depletion of the microbiota, CON-FMT, SD-FMT, SD + Mel-FMT, and V-FMT were provided drinking water containing 1 g/kg ampicillin (Santa Cruz Biotechnology, Dallas, TX, USA), 100 mg/kg gentamicin (Sigma-Aldrich), 0.5 g/kg neomycin (Sigma-Aldrich), 0.5 g/kg vancomycin (Hexal, Germany), and 10 mg/kg erythromycin (Sigma-Aldrich) for 10 days. A fresh antibiotic solution was prepared daily to ensure activity.

To prepare FMT material, fresh feces were collected from donor mice and immediately diluted with sterile PBS. PBS (1 mL) was used to dilute 50 mg of fecal pellets. Briefly, the stool was steeped in sterile PBS for approximately 15 min, shaken, and then centrifuged at 1000 rpm and 4 °C for 5 min. The supernatant was collected and centrifuged at 8000 rpm and 4 °C for 5 min. This supernatant was discarded, and the bacteria were retained and resuspended in PBS and filtered twice. The final bacterial suspension was mixed with an equal volume of 40% sterile glycerol to a final concentration of 20% and stored at –80 °C until transplantation [31]. For each mouse, 100 μL of bacterial suspension (10^8^ colony-forming unit [CFU]/mL) was transplanted into each recipient mouse by gavage each day for 14 consecutive days [32, 33]. Mice in the V-FMT group received an equal volume of sterile PBS containing 20% glycerol.

### *A. veronii* colonization experiment

To investigate the effects of *A. veronii* on cognitive impairment induced by SD, the mice were divided into three groups: the control (CON), *A. veronii* colonization (Aero), and *A. veronii* colonization supplemented with Mel (20 mg/kg) (A + Mel) groups (Fig. 1B). The Aero and A + Mel groups were provided with drinking water containing the same antibiotics used for FMT recipient mice for 10 days. Aero and A + Mel mice were then orally gavaged with 10^8^ CFU/mL of *A. veronii* in 0.1 mL PBS on the 11th day at 8 am. The CON group was orally gavaged with 0.1 mL PBS. In the A + Mel group, 20 mg/kg Mel was administered to mice via intraperitoneal injection 60 min before *A. veronii* colonization. CON and Aero mice were intraperitoneally injected with an equal volume of sterile saline containing 2% ethanol.

### LPS treatment experiment

To investigate the effects of LPS on cognitive impairment induced by SD, mice were divided into the control (CON), LPS (LPS), LPS supplemented with Mel (20 mg/kg) (LPS + Mel), and LPS supplemented with the TLR4 inhibitor TAK-242 (LPS + TAK-242) groups (Fig. 1C). The LPS-supplemented mice were administered intraperitoneal injections of LPS (27,840; Sigma, St. Louis, MO) 2 mg/kg every day, a single dose per day at 8:00 am. For treatment with Mel and TAK-242, 20 mg/kg Mel (LPS + Mel) and 150 mg/kg TAK-242 (LPS + TAK-242) were administered to mice by intraperitoneal injections 60 min before LPS supplementation as a single dose once per day for 3 days. Mice in the CON, LPS, and LPS + TAK-242 groups were intraperitoneally injected with an equal volume of sterile saline containing 2% ethanol.

### Butyrate treatment experiment

To investigate the effects of butyrate on cognitive impairment induced by SD, mice were divided into six groups: the SD (SD), SD + antibiotics (SD + Abs), SD + Mel supplementation (SD + Mel), SD + antibiotics + Mel supplementation (SD + Abs + Mel), SD + antibiotics + butyrate supplementation (SD + Abs + butyrate), and non-sleep-deprived control (CON) groups (Fig. 1D). The SD-treated mice were administered intraperitoneal injections of Mel 0 mg/kg (vehicle, SD, and SD + Abs) and 20 mg/kg (SD + Mel and SD + Abs + Mel) once, 60 min before SD, and a single dose per day at 7:00 am for a total of 3 days. For treatment with butyrate, 40 mM sodium butyrate (Sigma-Aldrich, St. Louis, MO, USA) was administered orally to mice by gavage (SD + Abs + Butyrate), 60 min before SD, and a single dose per day at 7:00 am for a total of 3 days. Mice in the CON, SD, SD + Abs, and SD + Abs + butyrate groups were intraperitoneally injected with an equal volume of sterile saline containing 2% ethanol. For substantial depletion of the microbiota, mice in the SD + Abs, SD + Abs + Mel, and SD + Abs + butyrate groups were provided drinking water containing the same antibiotics used for the FMT recipient mice for 10 days.

All mice were euthanized under anesthesia using 10% chloral hydrate after the experiment ended at 8:00 am on the final day. Hippocampal tissue, colonic contents, and fecal samples were collected. This experiment was repeated twice.

### Feces culture and colony-forming unit measurement

Feces were collected in tubes and diluted with a 10-weight volume of PBS. Each tube was vigorously vortexed and centrifuged for 10 min at 800 rpm. The supernatant was serially diluted 10^2^–10^8^ fold, and aliquots were streaked with a cell spreader on brain–heart infusion agar. After overnight incubation at 37 °C, CFUs were determined [34].

### Morris water maze

The Morris water maze (MWM) test was used to assess spatial learning and memory. The maze consisted of a round tank (120 cm in diameter, 50 cm in height) filled with warm water (23 ± 1 °C). Black nontoxic carbon ink was added to make the water opaque. The pool was divided into four quadrants (I, II, III, and IV). A moveable, hidden, circular platform was placed at a fixed location in quadrant IV and submerged approximately 1 cm below the water surface.

After acclimatization for 1 week, all mice were placed in a water pool without a platform for 1 min and allowed to swim. All experiments were performed at 8:00 a.m. To minimize the effects of stress on the experimental outcomes, behaviorally and physically healthy mice without any stereotypical characteristics were selected for further study. On the second day, each mouse was placed in water in all four quadrants in a fixed order to perform four training trials per day. The maximum trial duration was 60 s. Mice that failed to locate the hidden platform were manually guided. Once they reached the platform, they were allowed to remain there for 15 s. All mice received this training for five consecutive days. The mice were subjected to behavioral tests (training and detection periods) after FMT or *A. veronii* experiments. The mice in the butyrate and LPS supplementation groups were first subjected to behavioral training and then assessed after the test treatments.

MWM parameters included latency (s), path length (m), path velocity (mm/s) to reach the hidden platform, time spent in the target zone, and the number of crossings over the previous platform location when the platform was removed. The experiment was performed at the same time of the day, under the same environmental conditions. The animal movement was tracked using a computerized tracking system (XR-XM101; Shanghai Softmaze Information Technology Co., Ltd., China).

### Cell culture and treatment

BV2 immortalized murine microglia cells were maintained at 37 °C in a 5% CO_2_ humidified incubator in Dulbecco’s modified Eagle’s medium (Gibco, Franklin Lakes, NJ, USA) supplemented with 10% fetal bovine serum and 100 U/mL penicillin and streptomycin (Gibco). The cells were cultured in 12-well plates (5 × 10^5^ cells/mL). Some cells treated with 200 μM LPS were also treated with 5 mM butyrate (Sigma-Aldrich; LPS + butyrate). After butyrate supplementation for 30 min, some LPS + butyrate cells were sequentially treated with TLR4 inhibitor (10 μM TAK-242; MedChemExpress, Monmouth Junction, NJ, USA; LPS + TAK-242-cells), MCT1 inhibitor (10 nM AZD3965; MedChemExpress; LPS + butyrate + AZD3965), HDAC3 agonist (50 μM ITSA-1; MedChemExpress; LPS + butyrate + ITSA-1-cells), or nuclear factor-kappa B (NF-κB) antagonist (100 μM pyrrolidine dithiocarbamate [PDTC]; MedChemExpress; LPS + PDTC cells). Each plate of treated cells was incubated for 24 h. To generate conditioned media, BV2 cells treated with drug were grown in a serum-free growth medium for 24 h. Culture supernatants were collected, then added to HT-22 cells, and cultured in a humidified 5% CO_2_/95% air environment at 37 °C for 24 h. After BV2 microglia were treated under different conditions, culture supernatants were collected for ELISA analysis. BV2 cells were collected for western blotting for the detection of signaling pathway proteins (HDAC3, p-IκB, and p-P65). HT22 cells were collected for western blot analysis of cleaved caspase-3 levels. Each assay was repeated eight times.

### Western blot assay

The hippocampus tissues, BV2 cells, and HT-22 cells were rapidly isolated and lysed in RIPA lysis buffer (CW2333S; CWBIO, Beijing, China) containing 1% protease inhibitor cocktail (CW2200S; CWBIO, Beijing, China) and 1% phosphatase inhibitor cocktail (CW2383S; CWBIO, Beijing, China). The lysates were centrifuged at 14,000 × *g* for 15 min at 4 °C. The supernatants were collected, and the amount of protein was measured using a bicinchoninic acid kit (CW0014; CWBIO, Beijing, China), before the protein concentration was standardized. The protein samples were resolved using 10% sodium dodecyl sulfate–polyacrylamide gel electrophoresis (SDS-PAGE) and electro blotted onto a polyvinylidene fluoride membrane (Millipore; Billerica). Nitrocellulose membranes were blocked for 60 min using TBST (a mixture of Tris-buffered saline [TBS] and 0.05% Tween-20) containing 5% fat-free dry milk. They were then incubated in rabbit primary antibodies (TLR4, 1:1000, Abcam; cleaved caspase-3, 1:1000, CST; HDAC3, 1:1000; p-IκB,1:1000; p-P65, 1:1000; β-actin,1:8000, Abcam) overnight at 4 °C. After washing in TBST, they were incubated in horseradish peroxidase conjugated goat anti-rabbit IgG (1:5000; CW0103; CWBIO, Beijing, China) for 2 h at 37 °C. The protein bands were detected using an enhanced chemiluminescence kit (CW0049; CWBIO, Beijing, China). The protein band intensities were quantified using ImageJ software (version 1.4, National Institutes of Health, Bethesda). The protein level was normalized to the density ratio of β-actin, while the relative protein level in the CON group in vivo or in the control cells in vitro was defined as 100%. Each sample was assayed three times.

### Immunohistochemical staining

Paraffin sections were incubated in rabbit anti-Iba1 (1:500; ab178846; Abcam) primary antibody overnight at 4 °C. The sections were then rinsed in 0.01 M PBS (pH 7.4) and incubated in biotinylated goat anti-rabbit IgG (1:300, sc-2020; Santa Cruz) for 2 h at room temperature. After washing, the tissues were incubated in streptavidin–horseradish peroxidase (1:300; Vector Laboratories, Burlingame) for 2 h at room temperature. Immunoreactivity was visualized by incubating the tissue sections in 0.01 M PBS containing 0.05% DAB (Sigma) and 0.003% H_2_O_2_ for 10 min in the dark. The sections were then stained with hematoxylin and mounted. Control slides without the primary antibody were examined in all cases. Immunoreactive cells presented with yellow–brown staining in the cytoplasm. The localization and distribution of immunoreactive positive materials in the hippocampus were observed using a microscope (BX51; Olympus). For each mouse, representative coronal brain sections with similar coordinates (Bregma, 2.0 mm) were selected, five slices each from five animals/group were used in the analysis. ImageJ software (version 1.4, National Institutes of Health, Bethesda) was used to analyze the levels of microglia. Results are expressed as a mean integral optical density (IOD) of Iba1-positive cells, normalized to that in controls.

### Real-time reverse transcription-polymerase chain reaction (RT-PCR)

Mice feces sample DNA was extracted using the stool DNA Kit (DP328-02, TIANGEN) according to the manufacturer’s instructions. PCR amplification was performed using the AceQ qPCR SYBR green master mix (Q111-02; Vazyme Biotech, USA). Primers specific to 16S ribosomal RNA were used as an endogenous control to normalize loading between samples. The relative amount of 16S ribosomal DNA in each sample was estimated using the ΔΔCT. Primer sequences were designed using Primer-BLAST. RT-PCR primers sequences are as follows:

- *Aeromonas*: Fwd-5’GCGACTTCAAGCTGCAAGAG 3′, Rev 5’TTCAGTCGCTCGATGGTCTG 3’.
- *Bacteria 16S*: Fwd-5’TCCTACGGGAGGCAGCAGT 3’, Rev 5’GGACTACCAGGGTATCTAATCCTGTT 3’.

### Enzyme-linked immunosorbent assay (ELISA)

Hippocampus samples or culture supernatants of BV2 cells were collected for the detection of inflammatory factors (TNF-α, IL-6, IL-4, and IL-10) concentrations using a competitive ELISA (Uscn Life Science, Inc., Wuhan, China). The ELISA kits used to detect the levels of mouse LPS was purchased from Jianglai Industrial Limited By Share Ltd, Shanghai, China. All the tests were performed according to the manufacturer’s instructions. Each sample was tested in triplicate. The intra-assay coefficient of variation (CV) was < 10% and the inter-assay CV was < 12%.

### Gut microbiota analysis

Fresh colon contents were obtained from the mouse colon using 4 mL of sterile PBS. After centrifugation (1500 rpm, 5 min), the supernatant was discarded, and all samples were stored at − 80 °C until gut microbial analysis. Genomic DNA from the colon tissue of 21 mice was isolated using a PowerSoil DNA Isolation kit (MO BIO Laboratories, Carlsbad, CA, USA) according to the manufacturer’s instructions. The concentration and purity of isolated DNA were quantified using a Synergy HTX Multi-Mode Reader (Gene Company Ltd., Hong Kong, China). The V1-V9 region of the bacterial 16S rRNA gene was amplified using universal primers (27F: AGRGTTTGATYNTGGCTCAG; 1492R: TASGGHTACCTTGTTASGACTT) and purified with MagicPure® size selection DNA beads (TANGEN Biotech Corporation Ltd, Beijing, China). The abundance and diversity of the gut microbiota in mice were measured using the PacBio sequencing platform (Biomarker Technologies, Beijing, China). Circular consensus sequencing (CCS) was obtained from the raw subreads following minPasses ≥ 5 and minPredictedAccuracy ≥ 0.9 (SMRT Link version8.0). Then, 1200–1650 bp CCS was filtered into high-quality clean tags using Lima version 1.7.0, and chimera sequences were detected and removed using the UCHIME algorithm. Finally, the qualified sequences were clustered at a 97% similarity level using USEARCH (version 10.0), and 0.005% of the total sequences were identified as quality-filtered sequences to generate the operational taxonomic units (OTUs). Taxonomy was assigned to the OTUs using the SILVA database (v.123) with the RDP classifier at a 70% confidence threshold [35]. Alpha diversity indices, including the ACE, Chao1, Shannon, and Simpson indices, were calculated using QIIME2 software (Version 2020.8). Statistical significance between groups was determined by one-way analysis of variance (ANOVA). Beta diversity analysis was performed to investigate the structural variation in microbial communities across samples using binary Jaccard distance metrics and visualized via principal coordinate analysis (PCoA). Differences in the binary Jaccard distances among the groups were determined using analysis of similarities (ANOSIM). Linear discriminant analysis (LDA) effect size (LefSe) [36] was used to identify representative species. LDA was performed from the phylum to the genus level. LDA scores ≥ 3.0 and *p* < 0.05 were considered signature taxa and selected for plotting and further analysis. The Spearman rank correlation test was used (R package “psych”) to analyze the correlations between the signature microbial taxa and phenotypic variables. FDR adjusted *p* < 0.2 was considered statistically significant and visualized using the R package “corrplot”. Sequencing data were deposited in the Sequence Read Archive of the National Center for Biotechnology Information (Bioproject: PRJNA826223) for publication.

### Metabolomics profiling

Colon contents (100 µL) were added to 500 μL of extract containing an internal standard (1000:2; volume ratio of methanol to acetonitrile = 1:1; internal standard concentration 2 mg/L). The solutions were mixed by vortexing for 30 s. After centrifuging at 12,000 rpm for 15 min, the supernatant was collected for liquid chromatography–mass spectrometry (LC–MS) analysis. High-resolution mass spectral data were obtained using a UPLC Acquity I-Class PLUS system coupled with a Xevo G2-XS QTof (Waters, Wilmslow, UK) and Acquity UPLC HSS T3 (1.8 µm, 2.1 mm × 100 mm; Waters). As the chromatographic parameters, the mobile phase consisted of (A) aqueous 0.1% formic acid and (B) methanol with the addition of 0.1% formic acid; gradient program: 0 min (2% B), 0.25 min (2% B), 10 min (98% B), 13 min (98% B), 13.1 min (2% B), and 15 min (2% B); flow rate, 400 μL/min; injection volume, 1 μL. The MS parameters were set at an ion spray voltage of 2000 V (positive) and − 1500 V (negative), cone voltage of 25 V, ion source temperature of 150 °C, desolvation temperature of 500 °C, and desolvation gas flow of 800 L/h. The LC–MS/MS raw data were processed using MassLynx software (V4.2, Waters) and then imported into Progenesis QI software (version 2.3) for peak alignment and selection. Metabolites were identified using retention time, exact mass, and tandem MS data against METLIN and a self-built database (Biomarker Technologies Corporation, Beijing). Theoretical fragments were used for MS/MS identification.

### SCFA extraction and analysis

Fresh fecal contents (*n* = 8) samples were collected and stored at − 80 °C. Fecal samples were mixed with water and centrifuged. The supernatant was filtered and mixed with ether and sulfuric acid. After high-speed centrifugation, the ether layer was collected, and SCFA concentrations were measured using a model 6890 N gas chromatograph (Agilent, San Diego, CA, USA). The content is expressed as micrograms per milligram.

### Statistical analysis

The data were expressed as the mean ± standard error and analyzed using GraphPad Prism version 9 (GraphPad Software, La Jolla, CA, USA). Experiments were performed at least in five independent biological and at least two independent technical replicates. Differences between groups were analyzed using one-way ANOVA followed by Turkey’s multiple comparisons tests. All *p*-values < 0.05 were considered statistically significant.

## Results

### The gut microbiota mediates the neuroprotective effect of melatonin in neuroinflammation and memory impairment induced by SD

**Fig. 2:**
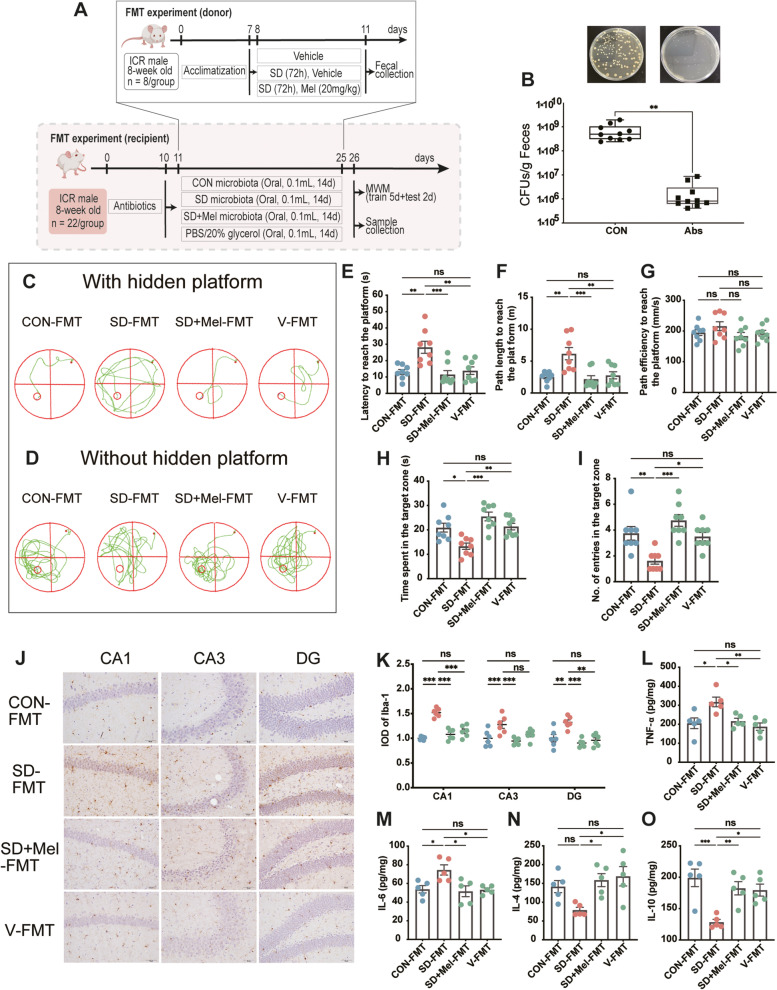
The gut microbiota mediated the neuroprotective effect of melatonin in memory impairment induced by sleep deprivation. **A** Schematic illustration of experimental design. **B** Comparison of bacterial colony-forming unit (CFU) in feces from control- and Abs-treated mice (*n* = 10). **C** Track plot of spatial memory test (with hidden platform). **D** Track plot of spatial memory test (without hidden platform). **E** Latency to reach the platform (*n* = 8). **F** Path length to reach the platform (*n* = 8). **G** Path efficiency to reach the platform (*n* = 8). **H** Time spent in the target zone (*n* = 8). **I** Number of entries into the target zone (*n* = 8). **J** Images of the immunohistochemical microglia in the different experimental groups. The immunohistochemical results were processed using ImageJ. Bar = 50 μm. **K** IOD of Iba1-positive cells in the hippocampal cornu ammonis (CA)1, CA3, and dentate gyrus (DG) regions (*n* = 6). **L–O** The levels of cytokines (TNF-α, IL-6, IL-4, and IL-10) in the hippocampus (*n* = 5). CON-FMT: receiving control microbiota FMT mice, SD-FMT: receiving sleep deprivation microbiota FMT mice, SD + Mel-FMT: receiving SD + Mel (20 mg/kg) microbiota FMT mice, V-FMT: receiving vehicle microbiota FMT mice. The data represent the mean ± SEM, *p* < 0.05 was set as the threshold for significance by one-way ANOVA followed by post hoc comparisons using Tukey’s test for multiple groups’ comparisons, *p < 0.05, **p < 0.01, ***p < 0.001

To test whether the neuroprotective effect of Mel on memory impairment induced by SD depends on the gut microbiota, we transplanted fecal microbiota from CON, SD, or SD + Mel groups into gut microbiota-depleted mice (which underwent 10 days of antibiotic pretreatment) for 2 weeks (Fig. 2A). The addition of antibiotics heavily depleted bacteria in the feces of the mice (Fig. 2B). Behavioral results showed that the latency was 115.8% higher in the SD-FMT group than in the CON-FMT group (*p* = 0.002), whereas the path length was 143.3% (*p* = 0.002) longer in the first test (with a hidden platform) (Fig. 2C, E–F). There was no significant difference in path efficiency among the groups (Fig. 2G). In the second test without the hidden platform (Fig. 2D), the number of entries into the target zone was 55.2% lower in the SD-FMT group than in the CON-FMT group (*p* = 0.005), whereas the time spent was 36.4% (*p* = 0.01) lower. Consequently, there was no significant difference between the SD + Mel-FMT and CON-FMT groups (Fig. 2D, H, I). The improved memory impairment of mice harboring microbiota from the SD + Mel donors compared to the SD donors suggests that the gut microbiota might contribute to the benefits conferred by Mel on cognitive dysfunction in SD mice (Fig. 2C–I).

To investigate whether neuroinflammation was induced by SD-derived gut microbiota, we evaluated changes in inflammatory factors and microglia immunohistochemical staining in the hippocampus. The integrated optical density (IOD) of Iba1-positive cells in the hippocampal cornu ammonis (CA)1, CA3, and dentate gyrus (DG) areas was 51.9% (*p* < 0.001), 27.6% (*p* = 0.01), and 32.3% (*p* = 0.002) higher in the SD-FMT group than in the CON-FMT group, respectively (Fig. 2J,K). We also observed significant increases in IL-6 (36.5%, *p* = 0.03) and TNF-α (47.2%, *p* = 0.01) levels and a significant decrease in IL-4 (49.6%, *p* = 0.04) and IL-10 (35.5%, *p* < 0.001) levels in the hippocampus of SD-FMT mice compared to CON-FMT mice (Fig. 2L–O). However, FMT of the “SD + Mel microbiota” significantly suppressed the activation of microglial cells, increased pro-inflammatory factors, and decreased anti-inflammatory factors. Excessive inflammation can cause cytotoxicity, interfere with cell growth, and induce apoptosis. Therefore, we determined the expression levels of cleaved caspase-3, Bax, and Bcl-2 using western blotting. Compared with the CON-FMT group, there was a significant downregulation in the expression of Bcl-2 (*p* = 0.021) and a significant upregulation in the expression of cleaved caspase-3 (*p* = 0.002) and Bax (*p* = 0.001) in the SD-FMT group. However, the FMT of the “SD + Mel microbiota” reversed these changes (Supplemental Fig. 1). The FMT experiments suggest that the gut microbiota is required for the protective effect of Mel on neuroinflammation, apoptosis, and memory impairment induced by SD.

### FMT treatment modulates gut microbiota composition in recipient mice

**Fig. 3:**
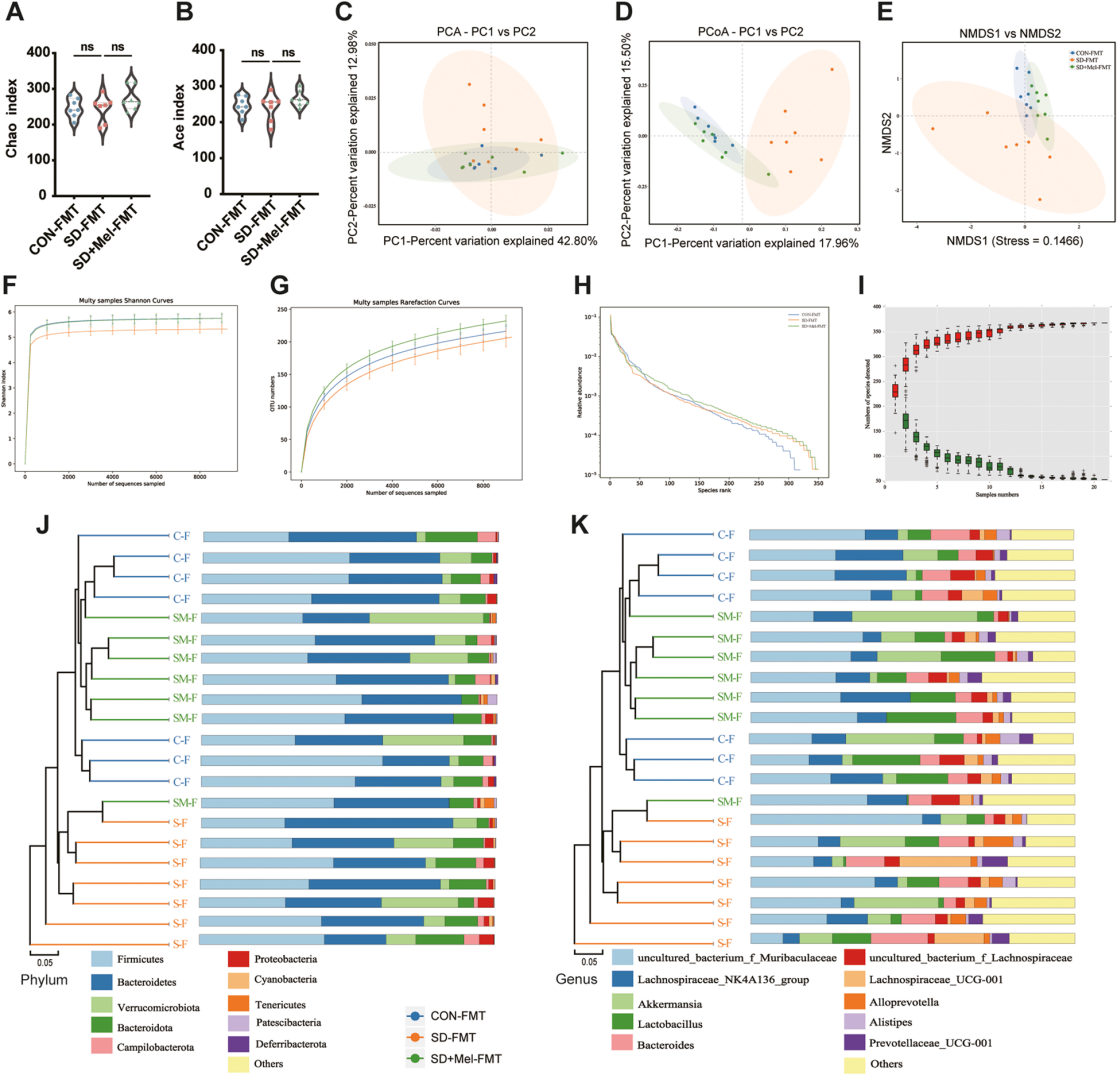
Composition of the colonic microbiota in FMT-treated mice. **A,B** Alpha diversity evaluation of colon microbial richness and evenness by measuring Chao and Ace diversity indexes. **C** Principal component analysis (PCA). **D** PCoA score plot. **E** Nonmetric multidimensional scaling (NMDS) score plot based on the binary_jaccard distance plot based on the OTU of the gut microbe. **F** Shannon curves. **G** OTU rank curves. **H** Rarefaction curves. **I** Rank abundance curve. **J** Unweighted pair-group method with arithmetic mean (UPGMA) analysis (at the phylum level). **K** Unweighted pair-group method with arithmetic mean (UPGMA) analysis (at the genus level) in the mice cecum of the CON-FMT, SD-FMT, and SD + Mel-FMT groups. CON-FMT: receiving control microbiota FMT mice, SD-FMT: receiving sleep deprivation microbiota FMT mice, SD + Mel-FMT: receiving SD + Mel (20 mg/kg) microbiota FMT mice. The data represent the mean ± SEM, *p* < 0.05 was set as the threshold for significance by one-way ANOVA followed by post hoc comparisons using Tukey’s test for multiple groups’ comparisons, *p < 0.05, **p < 0.01, ***p < 0.001

**Fig. 4:**
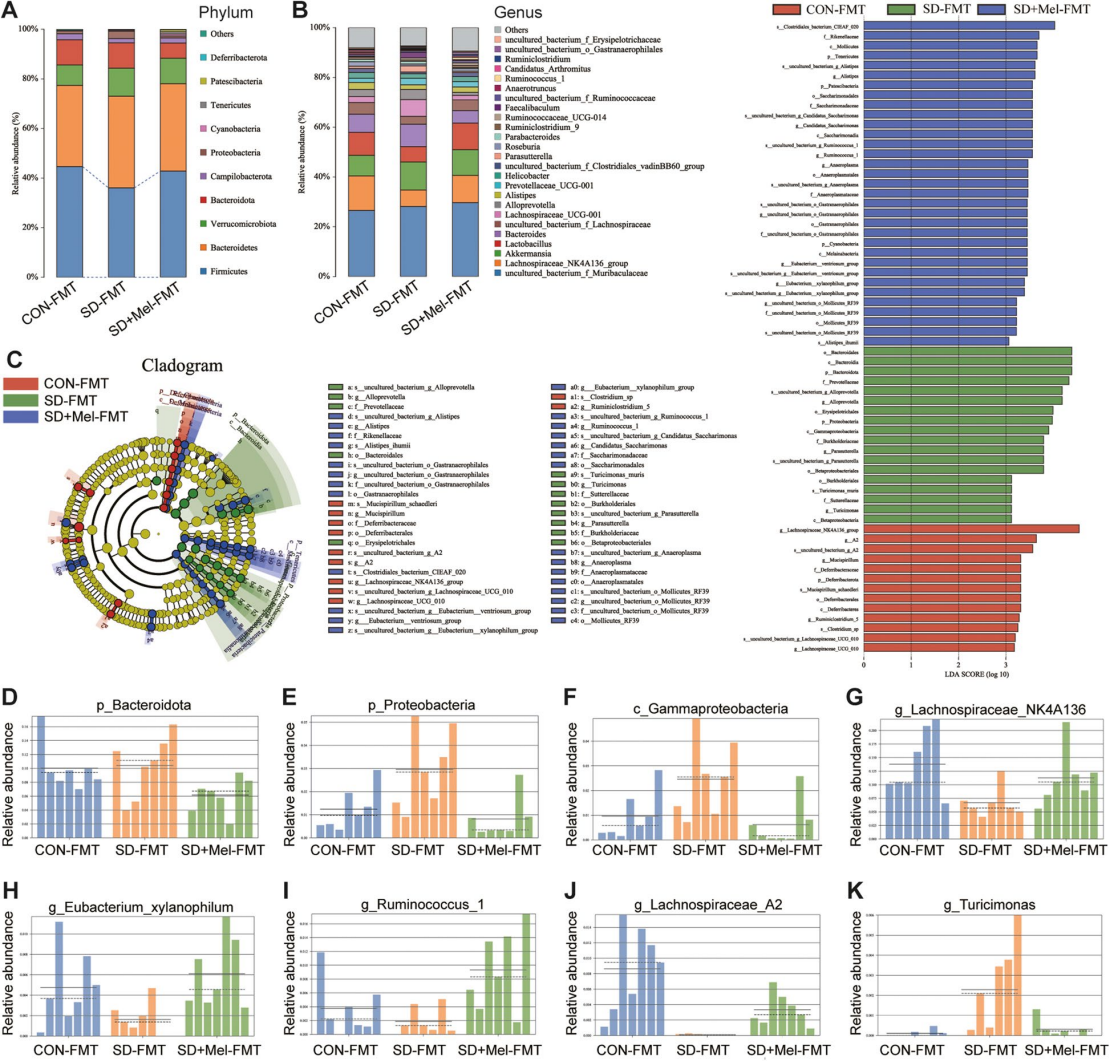
Composition and the key microflora of the colonic microbiota in FMT-treated mice. **A** Relative abundances of colonic microbiota at the phylum level in the 3 groups. **B** Relative abundances of gut microbiota at the genus level in the 3 groups. **C** Linear discriminant analysis effect size (LEfSe) was performed to identify the bacteria that are differentially represented between the different groups. **D–K** Relative abundance of *p_Bacteroidota*, *p_Proteobacteria*, *c_Gammaproteobacteria*, *g_Lachnospiraceae_NK4A136*, *g_Eubacterium_xylanophilum*, *g_Ruminococcus_1*, *g_ Lachnospiraceae_A2*, and *g_Turicionas* in the colon microbiota based on the LefSe results. Solid and dashed lines indicate the mean and median, respectively. CON-FMT: receiving control microbiota FMT mice, SD-FMT: receiving sleep deprivation microbiota FMT mice, SD + Mel-FMT: receiving SD + Mel (20 mg/kg) microbiota FMT mice

To test whether FMT modulated gut microbiota, we performed 16S rDNA gene sequencing to analyze the bacterial taxonomic composition following microbial therapy in recipient mice. A total of 21 samples were obtained from three groups (*n* = 7) of mice and subsequently sequenced to generate V1–V9 16S rRNA gene profiles. In total, 91,308, 91,002, and 90,919 raw reads were obtained for the CON-FMT, SD-FMT, and SD + Mel-FMT groups, respectively. There were 78,824, 77,428, and 77,628 clean tags in the CON-FMT, SD-FMT, and SD + Mel-FMT groups, respectively. Alpha diversity reflects the richness and diversity of the microbiota. There was no significant change in the Chao1, ACE, Simpson, and Shannon indices among the three treatment groups (*p* > 0.05) (Fig. 3A,B, Supplemental Fig. 2). To measure the degree of similarity between microbial communities, β-diversity was further evaluated using Bray–Curtis PCoA. The results showed a separation of each group (Fig. 3D), with 17.96%, 15.50%, and 9.66% variation explained by the principal components PC1, PC2, and PC3, respectively (Adonis, *p* = 0.001, R2 = 0.379). UPGMA results showed that the SD + Mel-FMT group was closer to the CON-FMT group than to the SD-FMT group, further validating the results of PCoA (Fig. 3J,K). At the phylum level, *Firmicutes*, *Bacteroidetes*, and *Verrucomicrobiota* were predominant (Fig. 4A). At the genus level, *uncultured_bacterium_f_Muribaculaceae, Lachnospiraceae_NK4A136_group*, *Akkermansia*, and *Lactobacillus* were the predominant microflora (Fig. 4B).

To identify the specific bacterial phyla associated with CON-FMT, SD-FMT, and SD + Mel-FMT groups, LDA and LEfSe were performed to identify the core taxa most likely to explain the differences between groups. As shown in Fig. 4C, *Bacteroidetes* and *Proteobacteria* were more abundant in the SD-FMT group than in the CON-FMT and SD + Mel-FMT groups (*Bacteroidetes*, *p* = 0.046, LDA score = 4.39; *Proteobacteria*, *p* = 0.012, LDA score = 3.98). Additionally, as shown in Fig. 4A, *Firmicutes* was decreased in the SD-FMT group compared to the CON-FMT and SD + Mel-FMT groups (*p* > 0.05). Furthermore, LEfSe analysis identified 64 taxa biomarkers in the three groups with an LDA score > 3 and *p* < 0.05. The relative abundances of *Lachnospiraceae_NK4A136_group* (*p* = 0.041, LDA score = 4.55), *Eubacteriumxylanophilum_group* (*p* = 0.027, LDA score = 3.39), *Ruminococcus_1* (*p* = 0.018, LDA score = 3.56), and *Lachnospiraceae_A2* (*p* = 0.001, LDA score = 3.64) were significantly lower in the SD-FMT group than in the CON-FMT and SD + Mel-FMT groups (Fig. 4G–J). In addition, the relative abundance of *Turicimonas* (*p* = 0.035, LDA score = 3.12) was significantly higher in the SD-FMT group than in the CON-FMT and SD + Mel-FMT groups (Fig. 4K), whereas there was no significant difference between the CON-FMT and SD + Mel-FMT groups (*p* > 0.05).

### FMT treatment modulates gut microbiota metabolite composition in recipient mice

**Fig. 5:**
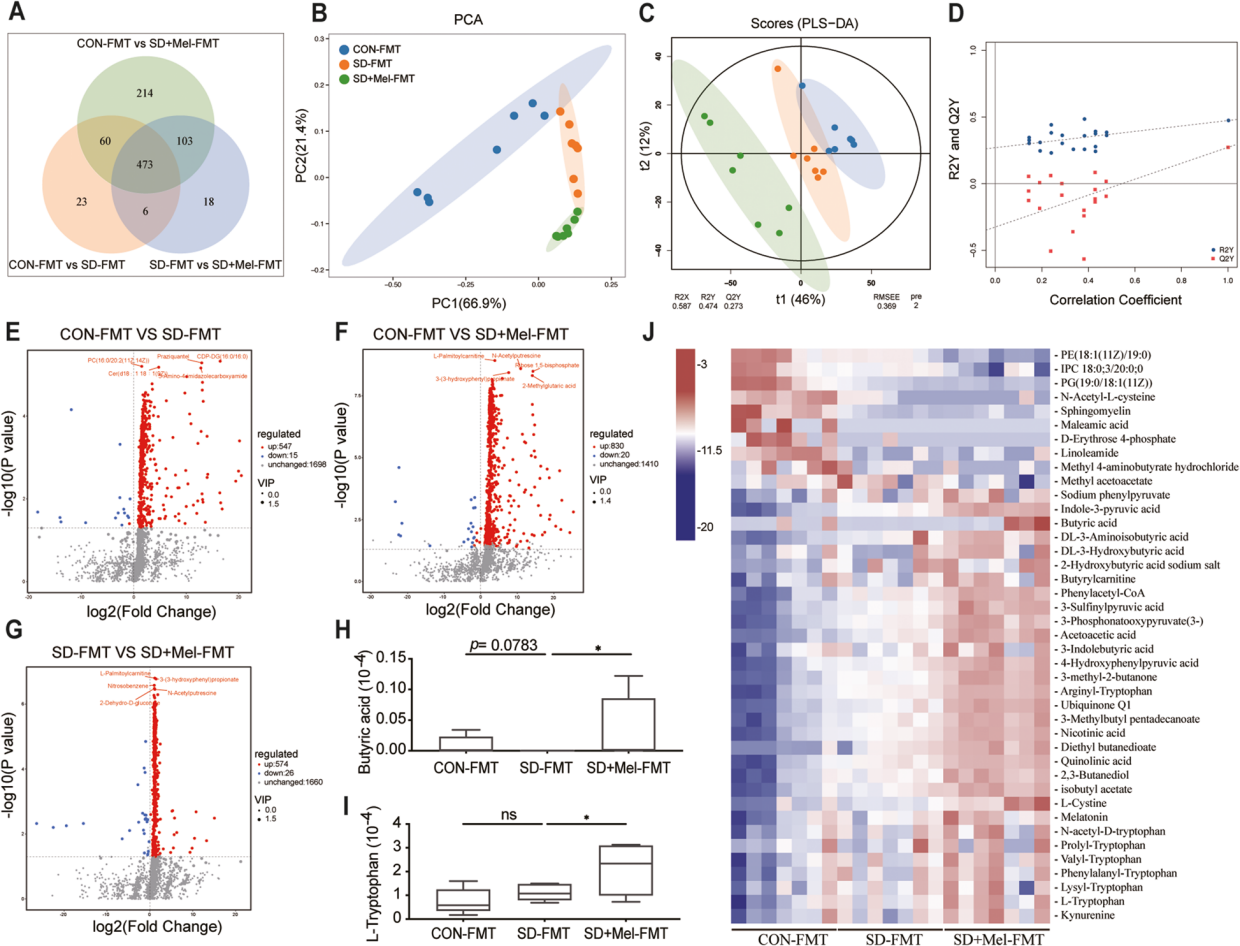
Composition of the colonic microbiota metabolites in FMT-treated mice. **A** Venn based on the microbiota metabolites. **B** β-diversity of principal component analysis (PCA). **C** Orthogonal projections to latent structures-discriminate analysis (OPLS-DA) score plot was performed on colon. **D** In the permutation validation plot the *Y*-axis intercepts of R2 and Q2 are 0.587 and 0.273, respectively, indicating that the model is valid. **E** Volcano plot based on the differential metabolite screening compared with the CON-FMT and SD-FMT groups. **F** volcano plot based on the differential metabolite screening compared with the CON-FMT and SD + Mel-FMT groups. **G** volcano plot based on the differential metabolite screening compared with the SD-FMT and SD-FMT groups. **H** The relative abundance of butyric acid. **I** The relative abundance of L-Tryptophan. **J** Heatmap showing the relative abundance of the key identified 41 metabolites (*p* < 0.05, VIP > 1). CON-FMT: receiving control microbiota FMT mice, SD-FMT: receiving sleep deprivation microbiota FMT mice, SD + Mel-FMT: receiving SD + Mel (20 mg/kg) microbiota FMT mice. The data represent the mean ± SEM, *p* < 0.05 was set as the threshold for significance by one-way ANOVA followed by post hoc comparisons using Tukey’s test for multiple groups’ comparisons, *p < 0.05, **p < 0.01, ***p < 0.001

Gut microbiota can influence the host due to its metabolites. Hence, we performed metabolomic profiling analyses on FMT recipient mice. Analysis of the metabolites showed that there were 2260 metabolites in the colon. A Venn diagram indicated that different treatments resulted in different metabolite changes (Fig. 5A). PCA analysis showed an obvious clustering of microbiota metabolite composition of all groups, which was closer to the CON-FMT group than to the SD-FMT group (Fig. 5B). To further verify the differences among samples from different groups, we applied orthogonal projections to latent structures discriminant analysis (OPLS-DA) to achieve this goal. The OPLS-DA model revealed a good separation among the three groups. The quality parameter values of the OPLS-DA model were predicted to be [R2X (cum) = 0.587, R2Y (cum) = 0.474], and fitness [Q2 (cum) = 0.273], indicating that the model had good reliability and predictability (Fig. 5C,D). The volcano plot indicated up- and downregulated differential metabolites based on statistical values (*p* < 0.05, | log2FC|> 1) (Fig. 5E–G). Specifically, compared to the CON-FMT group, 547 metabolites were upregulated and 15 downregulated in the SD-FMT group. However, 574 metabolites were increased and 26 metabolites were decreased in the SD + Mel-FMT group compared to the SD-FMT group. Furthermore, we screened the 41 most changed metabolites in the three groups (Fig. 5J). Compared to the SD-FMT group, the contents of butyric acid (*p* = 0.03) and L-tryptophan (*p* = 0.02) were significantly increased in the SD + Mel-FMT group (Fig. 5H,I).

### Correlation between microbiome composition and phenotypic variables

**Fig. 6:**
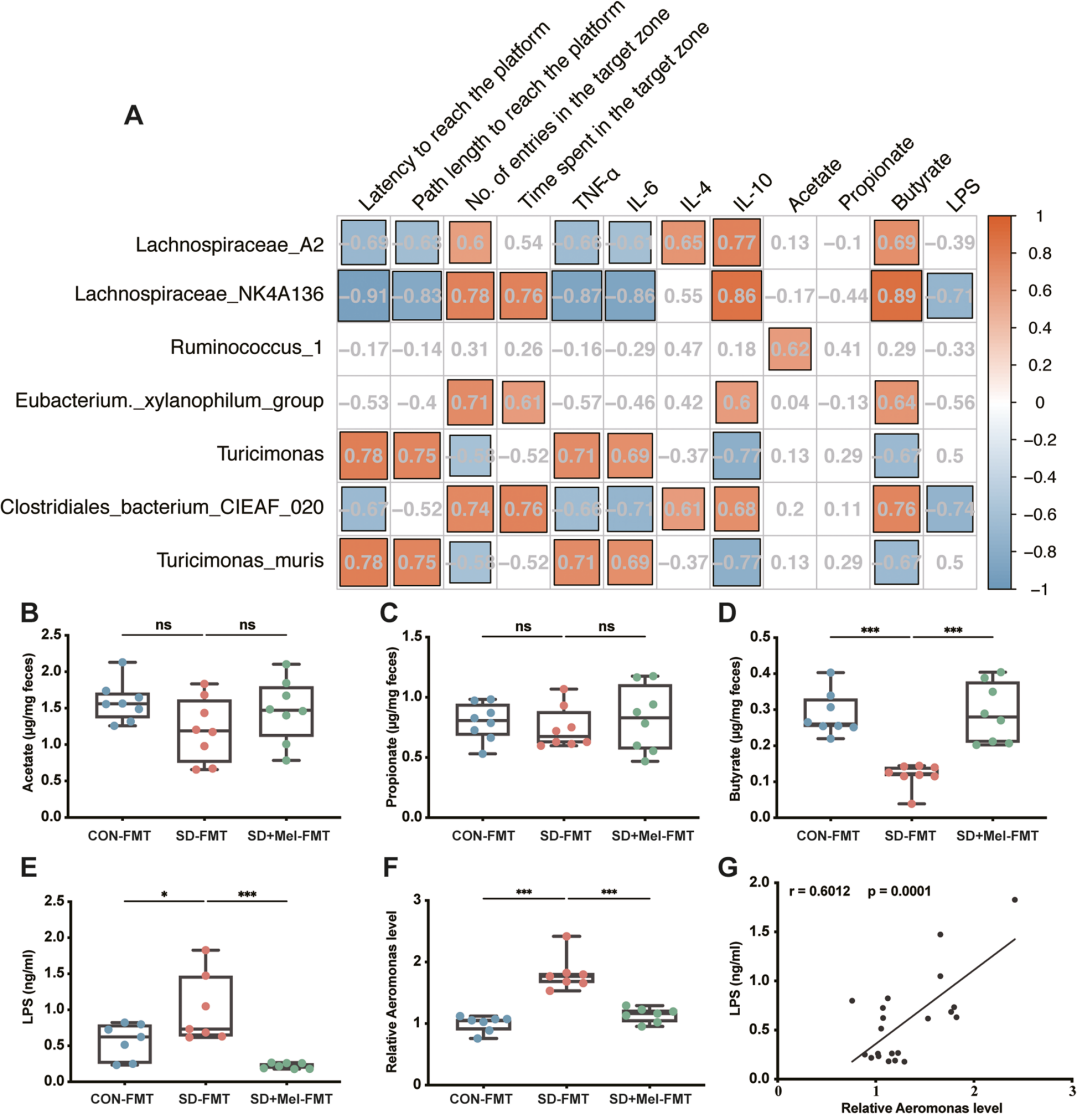
Correlation between the microbiome composition and phenotypic variables. **A** The correlation of top focus metabolites and the signature microbiota (Spearman correlation test, FDR < 0.05). **B** The contents of acetate in the feces (*n* = 8). **C** The contents of propionate in the feces (*n* = 8). **D** The contents of butyrate in the feces (*n* = 8). **E** The levels of LPS in the hippocampus (*n* = 7). **F** Relative abundance of colonic *Aeromonas* (*n* = 7). **G** Plots of correlation analysis between the fecal level of butyrate and colonic *Aeromonas*. *X*-axis represented the Relative abundance of colonic *Aeromonas*; *Y*-axis represented the fecal butyrate levels. CON-FMT: receiving control microbiota FMT mice, SD-FMT: receiving sleep deprivation microbiota FMT mice, SD + Mel-FMT: receiving SD + Mel (20 mg/kg) microbiota FMT mice. The data represent the mean ± SEM, *p* < 0.05 was set as the threshold for significance by one-way ANOVA followed by post hoc comparisons using Tukey’s test for multiple groups’ comparisons, *p < 0.05, **p < 0.01, ***p < 0.001

To further study whether metabolites were altered, we assayed fecal SCFA content by GC/MS. The results revealed a significant decrease in fecal butyrate in the SD-FMT group compared with that in the CON-FMT group (58.9%, *p* < 0.001). The SD + Mel-FMT group showed a significant increase in fecal butyrate compared to the SD-FMT group (146.8%, *p* < 0.001). There were no significant differences in the fecal levels of acetate and propionate among the three groups (Fig. 6B–D). Additionally, we assayed the relative abundance of *Aeromonas* in the colon using RT-PCR and the levels of LPS in the hippocampus using ELISA. A significant increase in LPS (76.8%, *p* = 0.017) and *Aeromonas* (64.0%, *p* < 0.001) was evident in the SD-FMT group compared with the CON-FMT group. The SD + Mel-FMT group showed a significant decrease compared with the SD-FMT group (Fig. 6E,F). Furthermore, correlation analysis showed that the relative abundance of *Aeromonas* was positively correlated with LPS levels (Fig. 6G). The fecal butyrate level, but not the other two SCFAs, was positively correlated with the microbial *g_Lachnospiraceae_NK4A136_group*, *g_Eubacterium_xylanophilum*, *g_ Lachnospiraceae_A2*, and *s_Clostridiales_bacterium_CIEAF_020* and negatively correlated with *g_Turicimonas* and *s_Turicimonas_muris*. These findings suggested that these microbial changes could be related to alterations in fecal butyrate levels (Fig. 6A). The microbial *g_Lachnospiraceae_NK4A136_group* was positively correlated with the time spent and the number of entries in the target zone and IL-10 and negatively correlated with the latency and path length to reach the platform, TNF-α, IL-6, and LPS (Fig. 6A). Thus, the gut microbial-mediated effect of SD or Mel may be correlated with its effect on regulating the potential of microbial metabolites (LPS and butyrate).

### Melatonin ameliorates the occurrence of neuroinflammation and memory impairment in mice induced by *A. veronii* colonization

**Fig. 7:**
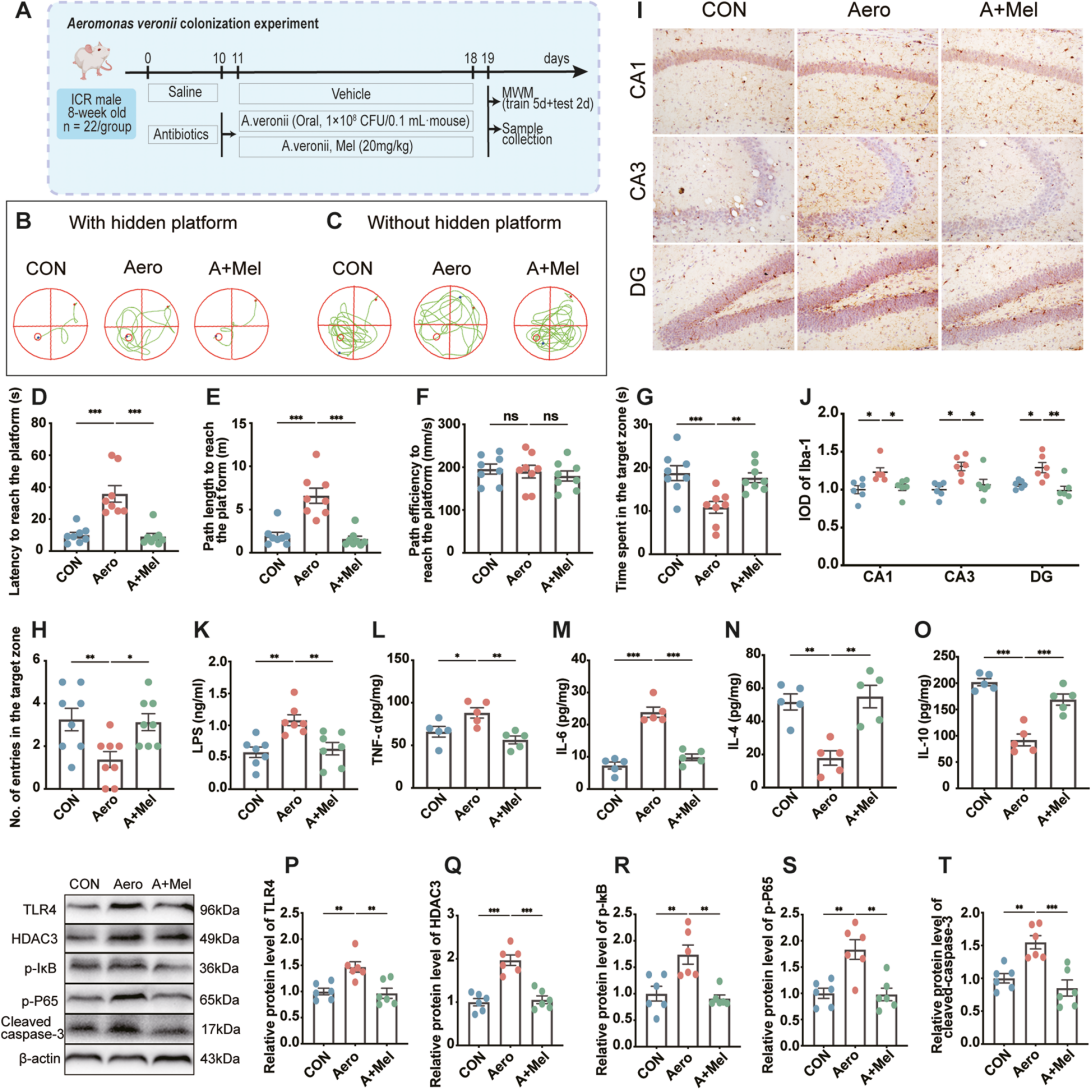
Melatonin ameliorates the occurrence of neuroinflammation and memory impairment in mice induced by *Aeromonas* colonization. **A** Schematic illustration of experimental design. **B** Track plot of spatial memory test (with hidden platform). **C** Track plot of spatial memory test (without hidden platform). **D** Latency to reach the platform (*n* = 8). **E** Path length to reach the platform (*n* = 8). **F** Time spent in the target zone (*n* = 8). **G** Number of entries into the target zone (*n* = 8). **H** Path efficiency to reach the platform (*n* = 8). **I** The levels of LPS in the hippocampus (*n* = 7). **J–M** The levels of cytokines (TNF-α, IL-6, IL-4, and IL-10) in the hippocampus (*n* = 5). **N** Images of the immunohistochemical microglia in the different experimental groups. The immunohistochemical results were processed using ImageJ. Bar = 50 μm. **O** IOD of Iba1-positive cells in the hippocampal cornu ammonis (CA)1, CA3, and dentate gyrus (DG) regions (*n* = 6). **P–T** Relative protein levels of TLR4, HDAC3, p-IκB, p-P65, and cleaved caspase-3 in the hippocampus (*n* = 6). CON: control group, Aero: *Aeromonas* colonization group, A + Mel: *Aeromonas* + melatonin (20 mg/kg) group. The data represent the mean ± SEM, *p* < 0.05 was set as the threshold for significance by one-way ANOVA followed by post hoc comparisons using Tukey’s test for multiple groups’ comparisons, *p < 0.05, **p < 0.01, ***p < 0.001

To verify the role of the *Aeromonas* level increase in SD-induced memory impairment, we established an *A. veronii* colonization mouse model (Fig. 7A). After *A. veronii* colonization, we observed an increase in latency (72.3%, *p* < 0.001) and path length (70.3%, *p* < 0.001) to reach the platform and a decrease in the time spent (42.2%, *p* = 0.001) and the number of entries (57.7%, *p* = 0.006) in the target zone of mice (Fig. 7B–H). Furthermore, the IOD of Iba1-positive cells in the hippocampal CA1, CA3, and DG areas was 23.1% (*p* = 0.017), 23.9% (*p* = 0.026), and 22.4% (*p* = 0.023) higher, respectively, in the Aero group than in the CON group (Fig. 7I, J). We also observed significant increases in LPS (86.8%, *p* = 0.002), IL-6 (69.4%, *p* < 0.001), and TNF-α (25.0%, *p* = 0.045) levels and a significant decrease in IL-4 (65.5%, *p* = 0.002) and IL-10 (54.4%, *p* < 0.001) levels in the hippocampus of the Aero group compared with the CON group (Fig. 7K–O). Increased expression levels of TLR4 (46.8%, *p* = 0.005, Fig. 7P), HDAC3 (96.9%, *p* < 0.001, Fig. 7Q), p-IκB (73.9%, *p* = 0.005, Fig. 7R), p-P65 (83.5%, *p* = 0.002, Fig. 7S), and cleaved caspase-3 (54.8%, *p* = 0.004, Fig. 7T) were observed in the Aero group compared to the CON group. However, Mel supplementation suppressed this process, resulting in no significant difference between the CON and A + Mel groups.

### Melatonin ameliorates LPS-induced neuroinflammation and memory impairment in mice

**Fig. 8:**
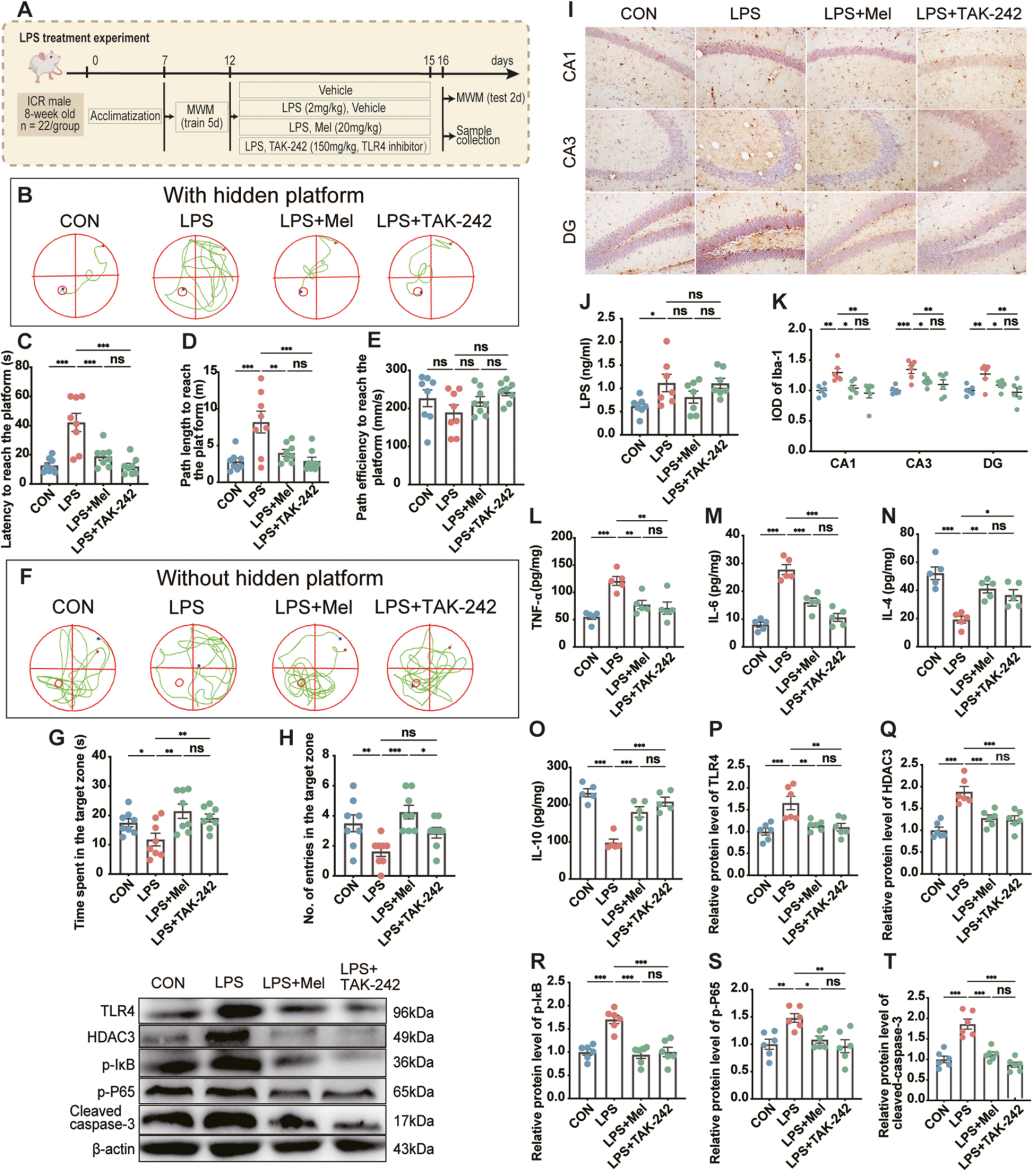
Melatonin ameliorates LPS-induced neuroinflammation and memory impairment in mice. **A** Schematic illustration of experimental design. **B** Track plot of spatial memory test (with hidden platform). **C** Latency to reach the platform (*n* = 8). **D** Path length to reach the platform (*n* = 8). **E** Path efficiency to reach the platform (*n* = 8). **F** Track plot of spatial memory test (without hidden platform). **G** Time spent in the target zone (*n* = 8). **H** Number of entries into the target zone (*n* = 8). **I** Images of the immunohistochemical microglia in the different experimental groups. The immunohistochemical results were processed using ImageJ. Bar = 50 μm. **J** The levels of LPS in the hippocampus (*n* = 7). **K** IOD of Iba1-positive cells in the hippocampal cornu ammonis (CA)1, CA3, and dentate gyrus (DG) regions (*n* = 6). **L–O** The levels of cytokines (TNF-α, IL-6, IL-4, and IL-10) in the hippocampus (*n* = 5). **P–T** Relative protein levels of TLR4, HDAC3, p-IκB, p-P65, and cleaved caspase-3 in the hippocampus (*n* = 6). CON: control group, LPS: lipopolysaccharides (2 mg/kg) group, LPS + Mel: LPS + melatonin (20 mg/kg) group, LPS + TAK-242: LPS + TAK-242 (TLR4 inhibitor, 150 mg/kg) group. The data represent the mean ± SEM, *p* < 0.05 was set as the threshold for significance by one-way ANOVA followed by post hoc comparisons using Tukey’s test for multiple groups’ comparisons, *p < 0.05, **p < 0.01, ***p < 0.001

To evaluate the relevance of Mel- and LPS-mediated memory impairment, we established an LPS-induced mouse model with or without Mel and TAK-242 supplementation (Fig. 8A). Compared with the CON group, the LPS-treated group showed an increase in latency (230.3%, *p* < 0.001; Fig. 8C) and path length (189.1%, *p* < 0.001; Fig. 8D) to reach the platform and a decrease in time spent (32.8%, *p* = 0.04; Fig. 8G) and the number of entries (53.6%, *p* = 0.005; Fig. 8H) in the target zone of mice. Furthermore, upregulation of LPS (66.6%, *p* = 0.013; Fig. 8J), TNF-α (54.4%, *p* = 0.001; Fig. 8L), and IL-6 (42.0%, *p* < 0.001; Fig. 8M) were observed. Downregulation of IL-4 (63.0%, *p* < 0.001; Fig. 8N) and IL-10 (57.8%, *p* < 0.001; Fig. 8O) was evident. Furthermore, the IOD of Iba1-positive cells in the hippocampal CA1, CA3, and DG areas was 29.5% (*p* = 0.01), 34.5% (*p* = 0.001), and 27.0% (*p* = 0.003) higher, respectively, in the LPS group than in the CON group (Fig. 8I, K). These results demonstrate an increase in the expression levels of TLR4 (65.6%, *p* = 0.001, Fig. 8P), HDAC3 (88.2%, *p* < 0.001, Fig. 8Q), p-IκB (70.3%, *p* < 0.001, Fig. 8R), p-P65 (48.0%, *p* = 0.007, Fig. 8S), and cleaved caspase-3 (86.0%, *p* < 0.001, Fig. 8T) in the LPS group compared to the CON group. These changes were reversed by Mel or TAK-242 supplementation, resulting in no significant differences between the groups.

### Effect of butyrate on melatonin improves memory impairment in SD mice

**Fig. 9:**
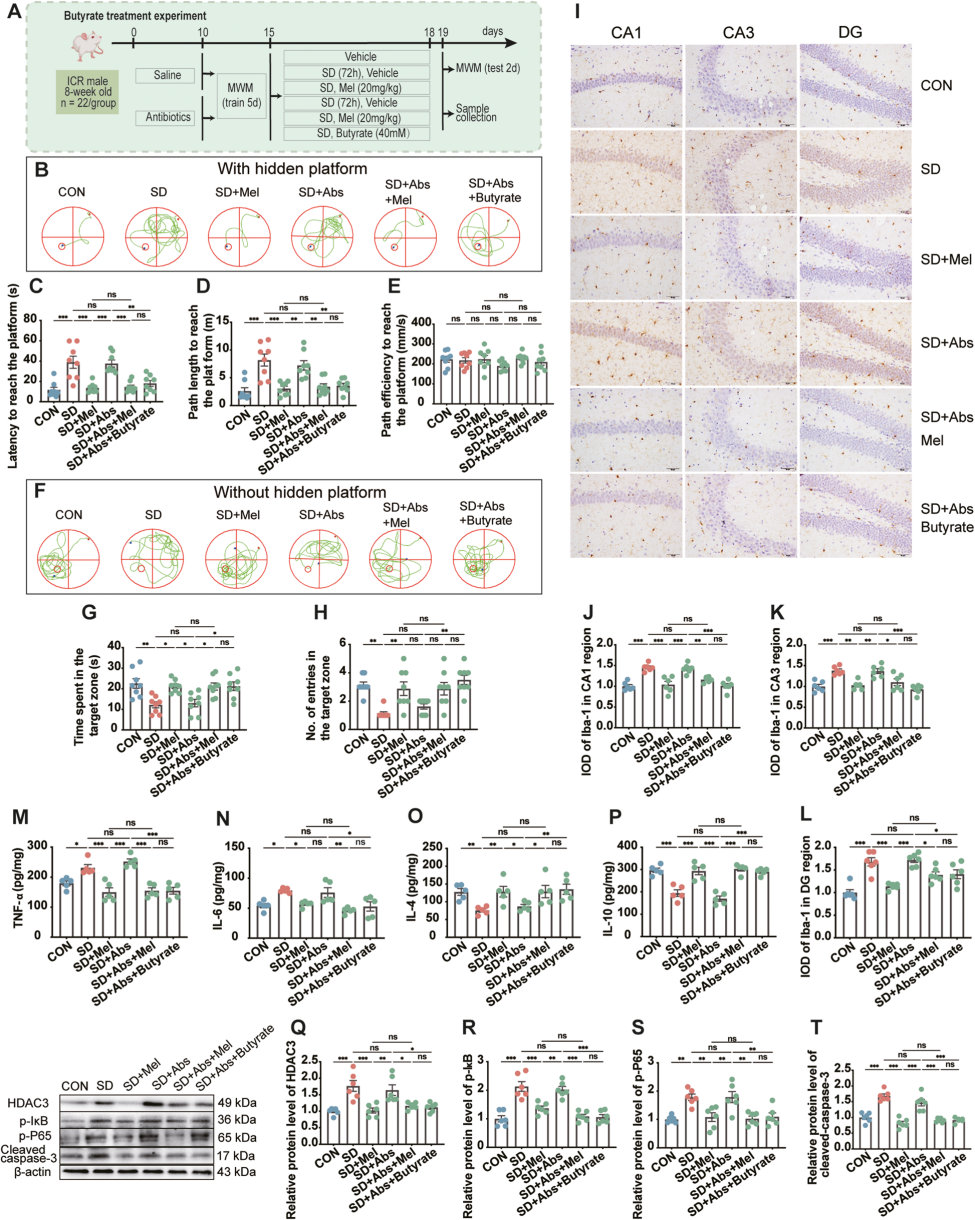
Effect of butyrate on Mel improved memory impairment in sleep-deprived mice. **A** Schematic illustration of experimental design. **B** Track plot of spatial memory test (with hidden platform). **C** Latency to reach the platform (*n* = 8). **D** Path length to reach the platform (*n* = 8). **E** Path efficiency to reach the platform (*n* = 8). **F** Track plot of spatial memory test (without hidden platform). **G** Number of entries into the target zone (*n* = 8). **H** Time spent in the target zone (*n* = 8). **I** Images of the immunohistochemical microglia in the different experimental groups. The immunohistochemical results were processed using ImageJ. Bar = 50 μm. **J–L** IOD of Iba1-positive cells in the hippocampal cornu ammonis (CA)1, CA3, and dentate gyrus (DG) regions (*n* = 6). **M–P** The levels of cytokines (TNF-α, IL-6, IL-4, and IL-10) in the hippocampus (*n* = 5). **Q–T** Relative protein levels of HDAC3, p-IκB, p-P65, and cleaved caspase-3 in the hippocampus (*n* = 6). CON: control group, SD: sleep deprivation group, SD + Mel: SD + melatonin (20 mg/kg) group, SD + Abs: SD + antibiotics group, SD + Abs + Mel: SD + antibiotics + Mel group, SD + Abs + Butyrate: SD + antibiotics + Butyrate (40 mM) group. The data represent the mean ± SEM, *p* < 0.05 was set as the threshold for significance by one-way ANOVA followed by post hoc comparisons using Tukey’s test for multiple groups’ comparisons, *p < 0.05, **p < 0.01, ***p < 0.001

To investigate whether butyrate could mediate the improved SD-induced cognitive impairment attributed to Mel, we observed changes in MWM when the platform was hidden or visible in mice in the CON, SD, SD + Abs, SD + Mel, SD + Abs + Mel, and SD + Abs + butyrate groups (Fig. 9A). Compared with the CON group, spatial memory impairment was evident in mice in the SD and SD + Abs groups. The latter two groups displayed a significant increase in path length and latency to reach the platform (*p* < 0.05; Fig. 9B–E) and a significant decrease in time spent and the number of entries in the target zone (*p* < 0.05; Fig. 9F–H). In contrast, Mel supplementation reversed the SD-induced changes in spatial memory impairment; no significant difference was observed in path length and latency to reach the platform, time spent, and the number of entries in the target zone among the SD + Mel, SD + Abs + Mel, and CON groups (Fig. 9B–H). Similar to Mel supplementation, butyrate supplementation improved SD-induced cognitive impairments. No difference was observed in any of the parameters between the SD + Abs + butyrate and CON groups (Fig. 9B–H). In addition, there was no significant difference in path efficiency among the groups (Fig. 9E). Thus, the results of the butyrate treatment experiment suggest that butyrate, as a signaling molecule of gut microbiota, may mediate the improvement of Mel in cognitive impairment caused by SD.

To investigate whether butyrate could mediate improved SD-induced neuroinflammation and apoptosis attributed to Mel, we examined the changes in the expression of Iba1 and the release of inflammatory cytokines and intracellular signaling proteins in the hippocampus. Compared with the CON group, neuroinflammation was evident in the SD and SD + Abs groups, which showed a significant increase in the IOD of Iba1-positive cells in the hippocampal CA1, CA3, and DG (*p* < 0.05; Fig. 9I–L) and IL-6 and TNF-α levels and a significant decrease in IL-4 and IL-10 levels (*p* < 0.05; Fig. 9M–P). For intracellular signaling proteins, there was an obvious upregulation in the expression of HDAC3 (*p* < 0.05; Fig. 9Q), p-IκB (*p* < 0.05; Fig. 9R), p-P65 (*p* < 0.05, Fig. 9S), and cleaved caspase-3 (*p* < 0.05, Fig. 9T) in the SD and SD + Abs groups compared to that in the CON group. Conversely, Mel supplementation reversed the changes in neuroinflammation and apoptosis caused by SD. The above indicators revealed no significant differences between the SD + Mel, SD + Abs + Mel, and CON groups. Similar to Mel supplementation, butyrate supplementation also improved SD-induced neuroinflammation and apoptosis. No difference was observed in any of the parameters between the SD + Abs + butyrate and CON groups.

### Effect of butyrate on inflammatory response and cell neurotoxicity in BV2 cells induced by LPS

**Fig. 10:**
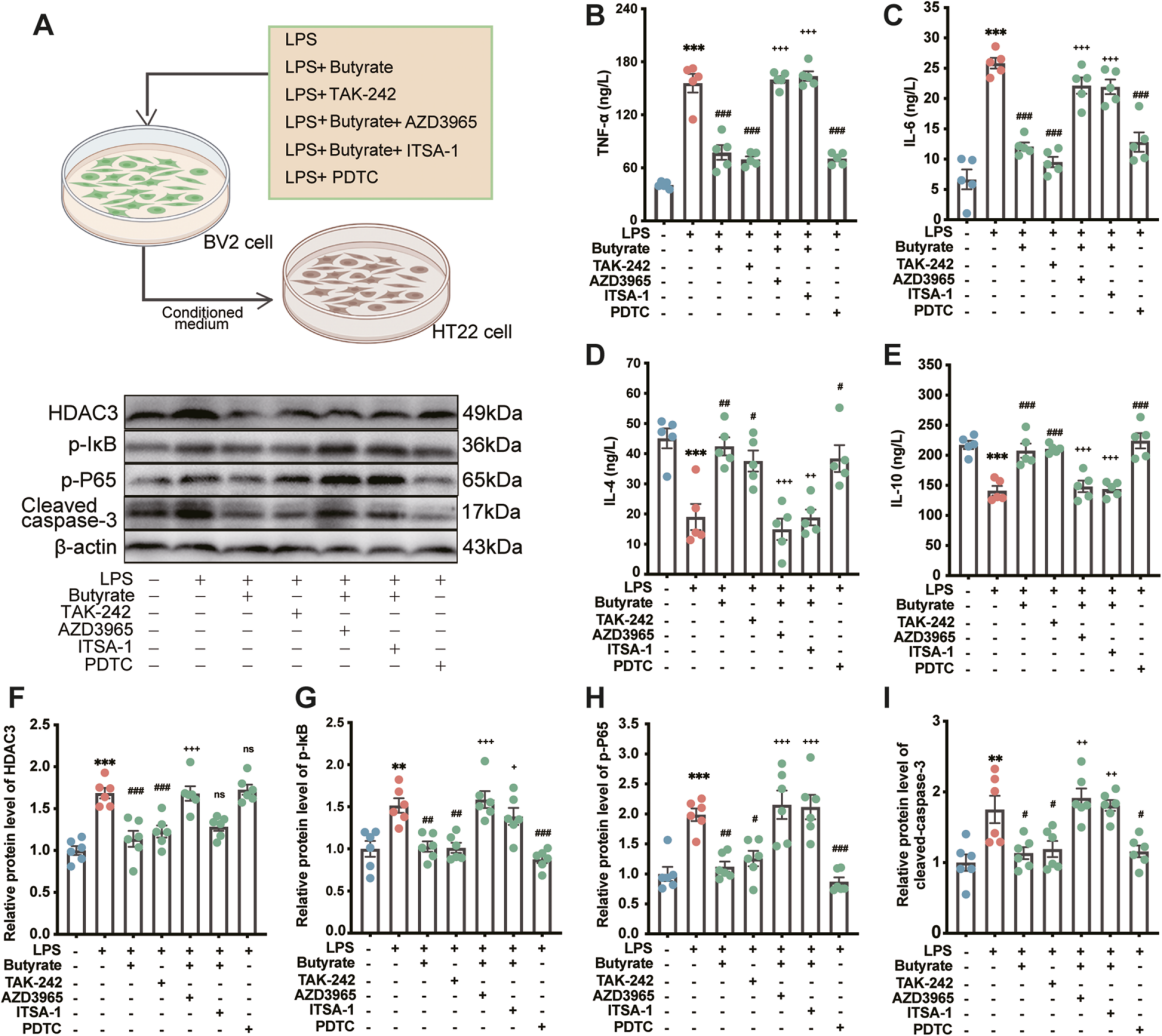
Effect of butyrate on inflammatory response and cell neurotoxicity in BV2 cells induced by LPS. **A** Schematic illustration of experimental design. **B–E** The levels of cytokines (TNF-α, IL-6, IL-4, and IL-10) in the BV2 cells (*n* = 5). **F–H** Relative protein levels of HDAC3, p-IκB and p-P65 in the BV2 cells (*n* = 6). **I** Relative protein levels of cleaved caspase-3 in the HT22 cells (*n* = 6). TAK-242: TLR4 inhibitor, PDTC:NF-κB antagonists, AZD3965:MCT1 inhibitor, ITSA-1: HDAC3 agonist. The data represent the mean ± SEM, *p* < 0.05 was set as the threshold for significance by one-way ANOVA followed by post hoc comparisons using Tukey’s test for multiple groups’ comparisons. *p < 0.05, **p < 0.01, ***p < 0.001 compared to the control group. # *p* < 0.05, ## *p* < 0.01, ### *p* < 0.001 compared to LPS group. + *p* < 0.05, +  + *p* < 0.01, +  +  + *p* < 0.001 compared to LPS + Mel group

To investigate the role of LPS or butyrate metabolite in Mel-induced cognitive impairment caused by SD, BV2 cells were treated with LPS to mimic neuroinflammation, with butyrate supplementation as an intervention (Fig. 10A). As expected, LPS exposure of BV2 cells led to increased secretion of TNF-α (283.8%, *p* < 0.001, Fig. 10B) and IL-6 (288.7%, *p* < 0.001, Fig. 10C) and decreased secretion of IL-4 (57.8%, *p* = 0.001, Fig. 10D) and IL-10 (35.0%, *p* < 0.001, Fig. 10E). Treatment with LPS significantly induced an increase in the relative expression levels of HDAC3 (68.5%, *p* = 0.001, Fig. 10F), p-IκB (51.4%, *p* = 0.001, Fig. 10G), and p-P65 (98.7%, *p* < 0.001, Fig. 10H) proteins in BV2 cells compared to the control group. In addition, we employed a microglia-conditioned medium (CM) system to evaluate whether the alleviation of microglial neurotoxicity by butyrate is involved in the survival of neural cells. CM derived from LPS‐induced BV2 microglia, with or without butyrate pretreatment, was added to HT22 cells. LPS-induced CM stimulated HT22 cell apoptosis by upregulating cleaved caspase-3 levels (42.9%, *p* = 0.002, Fig. 10I). However, butyrate pretreatment effectively reversed these LPS-induced changes. In contrast, after treatment with TAK-242, we observed downregulation of HDAC3 (27.2%, *p* = 0.001, Fig. 10F), p-IκB (33.2%, *p* = 0.001, Fig. 10G), and p-P65 proteins (36.7%, *p* < 0.001, Fig. 10H) compared to the LPS group. Our results suggest that inhibition of MCT1 by AZD3965 resulted in the upregulated expression of HDAC3 (47.4%, *p* = 0.001, Fig. 10F), p-IκB (54.3%, *p* = 0.001, Fig. 10G), and p-P65 proteins (92.1%, *p* < 0.001, Fig. 10H) in the LPS + butyrate + AZD3965-treated group compared to the LPS-treated group. Furthermore, after treatment with ITSA-1 as an HDAC3 agonist, upregulation of p-IκB (34.8%, *p* = 0.001, Fig. 10G) and p-P65 proteins (88.7%, *p* < 0.001, Fig. 10H) was evident compared to the LPS + butyrate group. There was no effect on the expression level of HDAC3 protein. However, treatment with PDTC (an NF-κB antagonist) imitated the beneficial effect of butyrate.

## Discussion

Sleep loss is a stressor that affects multiple tissues in the body. Pre-laboratory studies documented both cognitive impairment and intestinal dysfunction in sleep-deprived mice and demonstrated that exogenous Mel was effective in alleviating SD-induced impairment [2, 11]. Dysbiosis of the gut microbiota leads to impairment of brain functions, such as memory formation and cognitive function [37, 38]. We hypothesized that there is close communication between the gut and brain in sleep-deprived mice. To explore this hypothesis, we established an FMT model to further verify the role of gut microbes in memory impairment caused by SD. We treated mice with antibiotics to deplete most of the intestinal microbiota and facilitate subsequent colonization by fecal bacteria [39]. Fecal bacteria of mice in the CON, SD, and SD + Mel groups were transplanted into the recipient mice. We found that recipient mice colonized with the SD microbiota exhibited increases in latency and path length to reach the platform and decreases in the number of entries and time spent in the target zone, indicated that microbial dysbiosis caused by SD could influence cognitive impairment. Similar to our findings, mice receiving gut microbiota from Parkinson’s disease exhibited both gastrointestinal dysfunction and motor deficits. In addition, dopaminergic neuronal death has been detected in the SN of recipients [40]. Similarly, we also found that recipient mice that received the SD microbiota displayed an increased number of Iba1-positive microglia, upregulation of pro-inflammatory factors (IL-6 and TNF-α), and downregulation of inflammatory factors (IL-4 and IL-10) in the hippocampus. Further analysis showed that the protein levels of Bax and cleaved caspase-3 increased and that of Bcl-2 decreased in the hippocampus of SD-FMT mice. These results indicate the presence of neuroinflammatory responses and neuronal loss in the hippocampus of SD-FMT mice, which is consistent with the phenotype of SD-induced brain injury. However, recipient mice colonized by the SD + Mel microbiota did not show obvious cognitive impairment, and over-activated microglia, neuroinflammatory responses, and apoptosis were not observed in the hippocampal region. These results suggest that Mel could reverse the imbalance of intestinal microbiota induced by SD, and the improved intestinal microbiota does not affect the memory function of normal mice. These results indicated that changes in the gut microbiota and the resulting harmful symptoms of SD can be transmitted.

An increasing number of studies have confirmed that intestinal microbial communities can also affect the cognitive function of animals via the gut–brain axis [41]. Further microbiota analysis suggested that the protective effects of Mel treatment might be mediated by the reconstruction of the normal gut microbiota. In general, the present alpha- and beta diversity results revealed similar microbial communities in the SD + Mel-FMT and CON-FMT groups. In addition, comparisons at various taxon levels between the CON-FMT and SD + Mel-FMT groups revealed no significant differences in gut microbiota profiles, suggesting that the administration of Mel restored the healthy microbiota in SD-induced mice.

In the FMT experiments, our targeted assays revealed that the relative abundance of *Aeromonas* in the SD-FMT group was significantly upregulated relative to the CON-FMT group. This finding was consistent with the reported alterations in *Aeromonas* in sleep-deprived mice [11]. In contrast, the relative abundance of *Aeromonas* in the SD + Mel-FMT group was significantly lower than that in the SD-FMT group. *Aeromonas* is a gram-negative genus of bacteria belonging to the phylum *Proteobacteria*, class *Gammaproteobacteria*, order *Aeromonadales*, and family *Aeromonadaceae* and is a clinically important human pathogen that causes intestinal and parenteral infections, and its cell wall component LPS has been shown to cross the intestinal barrier and enter the systemic circulation by stimulating the permeability of the BBB, causing neuroinflammation [42, 43]. A previous study documented *Aeromonas* neurotoxicity on developmental motor reflexes and brain oxidative stress in the offspring of mice [44, 45]. In addition, we observed a significant upregulation of *Proteobacteria* and *Gammaproteobacteria* in SD-FMT mice. Therefore, we speculate that *Aeromonas* may mediate acute SD-induced cognitive impairment in mice. In the present study, mice colonized with *A. veronii* exhibited impaired SD-like cognitive function and significantly increased LPS levels in the hippocampus. Further tests revealed an increased neuroinflammatory response and apoptosis in the hippocampus, as evidenced by activation of microglia, increased pro-inflammatory cytokines, decreased anti-inflammatory cytokines, and increased levels of cleaved caspase-3, Mel supplementation ameliorated the changes in these indicators. The collective findings suggest that Mel can improve memory impairment caused by *Aeromonas* colonization.

The foregoing results suggest that *Aeromonas* mediates SD-induced cognitive dysfunction in mice. We also observed elevated levels of LPS in the hippocampus of SD-FMT mice. Correlation analysis showed that the relative abundance of colonic *Aeromonas* was positively correlated with the amount of LPS in the hippocampus. It has been reported that in the case of bacteriophage, LPS destroys the stability of the intestinal barrier and enters the systemic circulation. On the one hand, it reaches multiple tissues of the body through blood circulation, and on the other hand, it can activate immune cells to release a large number of inflammatory factors, aggravating the occurrence of systemic inflammation [46, 47]. Circulating LPS and the released inflammatory factors act to destabilize and increase the permeability of the BBB. The LPS can then breach the BBB and enter the brain parenchyma [48]. Thus, we hypothesized that LPS mediates SD-induced cognitive dysfunction in mice. In support of this hypothesis, normal mice treated with LPS exhibited SD-like cognitive impairment and a significant increase in LPS content was observed in the hippocampus, accompanied by hyperactivation of microglia and a large release of pro-inflammatory factors. Similar to our results, high levels of LPS were also previously detected of mice receiving FMT from sleep-deprived individuals [12]. Further mechanistic studies revealed that the expression levels of TLR4, p-P65, p-IκB, and cleaved caspase-3 in the hippocampal cells of *A. veronii*-colonized and LPS-treated mice were significantly increased. Exogenous Mel supplementation effectively reversed these changes. These collective results suggest that Mel alleviates hippocampal neuroinflammation induced by *Aeromonas* and LPS, ultimately ameliorating SD-like cognitive impairment in mice.

We observed that healthy microflora was restored in SD-induced mice after Mel administration; the resulting microflora was similar to that in the control group. Interestingly, we observed a large increase in butyrate-producing bacteria in recipient mice transplanted with SD + Mel microbes, including the *Lachnospiraceae_NK4A136_group*, *Eubacteriumxylanophilum*, *Ruminococcus_1*, and *Lachnospiraceae_A2*. A previous study found that the abundance of *Lachnospiraceae_NK4A136_group* can improve the intestinal barrier function of aging rats. This group comprises one of the main butyrate-producing bacteria, and its abundance is significantly negatively correlated with the level of inflammation [49, 50]. In addition, Eubacterium species, such as *E. rectale* and *E. eligens*, have been positively associated with several markers of lower frailty, improved cognitive ability, and increased production of SCFA and branched-chain fatty acid. *Eubacterium* spp. also showed negative correlations with inflammatory markers, including IL-2 and C-reactive protein [51]. *Ruminococcus_1* was also positively correlated with a reduction in depression-like behavior [52]. We also found using GC–MS that the metabolites between different groups changed significantly, similar to the changes in microorganisms. In this study, 574 metabolites were significantly increased and 26 metabolites were decreased in the SD + Mel-FMT group compared with the SD-FMT group. Among them, butyric acid and L-tryptophan were significantly upregulated metabolites in the SD + Mel-FMT group. Further LC–MS analysis showed that butyrate content decreased significantly in the feces of SD-FMT mice, but no changes were observed in acetate and propionate content. However, transplantation of microbiota from SD + Mel mice significantly restored the SD-induced reduction of butyrate. This finding suggests that increased microbial butyrate production could play a dominant role in mediating the gut microbiota-related effect of Mel on cognitive impairment caused by SD.

Importantly, butyrate has received the most attention among SCFAs as a key mediator of anti-inflammatory activity [53]. A recent study demonstrated that oral administration of butyrate to Alzheimer’s mice improved neuroinflammation and cognitive impairment [54]. In the present study, butyrate or Mel was administered to sleep-deprived mice to further verify the beneficial effects of butyrate. The results showed that supplementation with butyrate or Mel effectively alleviated the number of Iba1-positive cells in the hippocampus of sleep-deprived mice. The levels of pro-inflammatory cytokines and pro-apoptotic proteins increased significantly, ultimately reversing cognitive function in the mice. However, our previous study indicated that supplementation of SD mice with Mel (SD + Mel and SD + Abs + Mel groups) significantly increased the levels of Mel and butyrate, whereas supplementation with butyrate (SD + Abs + butyrate group) only restored butyrate content and did not eliminate the suppression of Mel secretion caused by SD [55]. These observations suggest that butyrate, as a signal molecule of the gut–brain axis, can mediate the improvement effect of Mel on SD-induced memory impairment.

Microglia are innate immune cells in the brain that function as crossroads in the regulation of immune responses in the brain [56, 57]. LPS entering the brain binds to TLR4 receptors on microglia, initiates downstream IκB/NF-κB or mitogen-activated protein kinase/extracellular signal activated kinase (MAPK/ERK) signal transduction pathways via MyD88, activates related proteins, and promotes the pro-inflammatory factors TNF-α and IL-6. Abundant secretion then produces a strong inflammatory effect [58, 59]. In the brain, butyrate can pass through the cell membrane into the cell through the transporter and exert an anti-inflammatory effect by inhibiting histone deacetylase. In contrast, butyrate produces an anti-inflammatory effect by binding to receptors and activating downstream signaling pathways [60, 61]. The MCT1 transporter is widely expressed in microglia, whereas G protein-coupled receptors are poorly expressed in microglia [62]. Therefore, we speculate that butyrate and LPS, two metabolites closely related to inflammation, may use microglia as target cells to regulate the inflammatory response in the brain. BV2 cells exposed to LPS-treated BV2 cell culture medium displayed an increased secretion of pro-inflammatory cytokines, decreased secretion of anti-inflammatory cytokines, and increased content of HDAC3, p-IκB, and p-P65 compared to the control group. In HT22 cells, the increase of cleaved caspase-3 protein suggested the occurrence of apoptosis. However, butyrate treatment reversed the LPS-induced changes. Furthermore, the addition of the TLR4 inhibitor TAK-242 and the NF-κB antagonist PDTC mimicked the ameliorative effect of butyrate on LPS-induced inflammatory responses in BV2 cells. Supplementation with the MCT1 inhibitor AZD3965 and the HDAC3 agonist ITSA-1 blocked the protective effect of butyrate.

## Conclusions

**Fig. 11:**
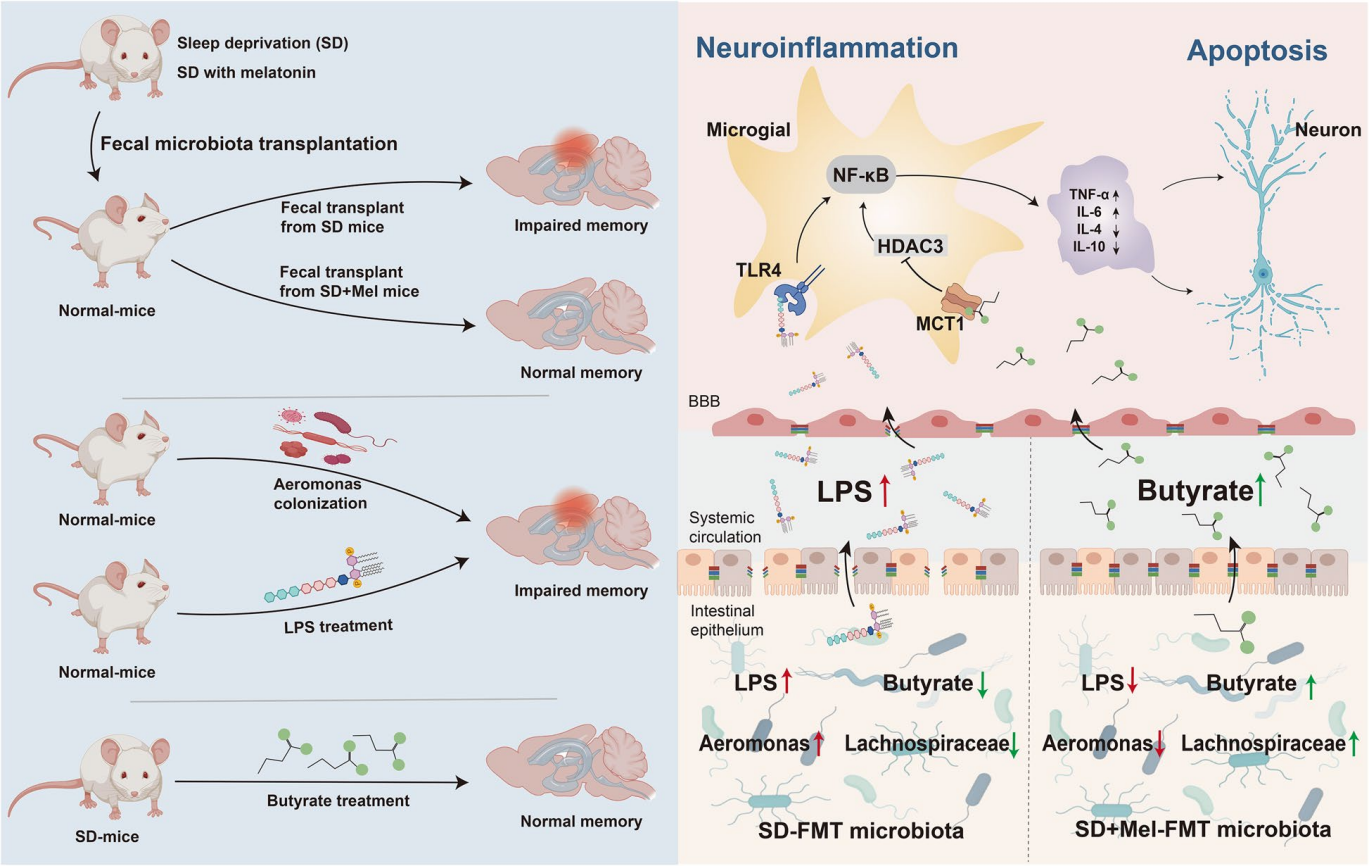
Schematic diagram of the protective effects of melatonin on cognitive impairment caused by sleep deprivation through the microbiota–gut–brain axis. Briefly, gut microbes and their metabolites mediate the ameliorative effect of melatonin on SD-induced cognitive impairment. A feasible mechanism is that Mel downregulates the *Aeromonas* population and production of the constituent LPS production and upregulates the *Lachnospiraceae_NK4A136* population and the production of the butyrate metabolite by remodeling gut microbiota homeostasis. These events inhibit the TLR4/HDAC3/NF-κB signaling pathway, thereby preventing neuroinflammation and ultimately alleviating neuronal apoptosis and memory impairment in sleep-deprived mice. HDAC3: histone deacetylase3, LPS: lipopolysaccharide, Mel: melatonin, NF-κB: nuclear factor-κB, SD: sleep deprivation, TLR4: Toll-like receptor 4

The present data reveal the protective effects of Mel on SD-induced cognitive impairment. Further mechanistic studies demonstrate that downregulation of the levels of *Aeromonas* and LPS and upregulation of the levels of *LachnospiraceaeNK4A136* and butyrate could constitute an underlying mechanism responsible for the neuroprotective effects of Mel in cognitive impairment caused by SD (Fig. 11).

## Supplementary Information

**Additional file 1:** **Supplemental Figure 1.** The gut microbiota mediated the neuroprotective effect of melatonin in neuronal apoptosis induced by sleep deprivation. (A-D) Relative protein levels of Bcl-2, Bax and cleaved caspase-3 in the hippocampus (*n* = 6). CON-FMT: receiving control microbiota FMT mice, SD-FMT: receiving sleep deprivation microbiota FMT mice, SD+Mel-FMT: receiving SD+Mel (20 mg/kg) microbiota FMT mice. The data represent the mean ± SEM, *p* < 0.05 was set as the threshold for significance by one-way ANOVA followed by post hoc comparisons using Tukey’s test for multiple groups’ comparisons, *p < 0.05, **p < 0.01, ***p < 0.001. **Supplemental Figure 2.** Composition of the colonic microbiota in FMT-treated mice. (A) OTU number, (B) Simpson index, (C) Shannon index. CON-FMT: receiving control microbiota FMT mice, SD-FMT: receiving sleep deprivation microbiota FMT mice, SD+Mel-FMT: receiving SD+Mel (20 mg/kg) microbiota FMT mice. The data represent the mean ± SEM, *p* < 0.05 was set as the threshold for significance by one-way ANOVA followed by post hoc comparisons using Tukey’s test for multiple groups’ comparisons, *p < 0.05, **p < 0.01, ***p < 0.001. **Supplemental Figure 3.** The levels of LPS in the hippocampus (n = 7). CON: control group, SD: sleep deprivation group, SD + Mel: SD + melatonin (20 mg/kg) supplement group. The data represent the mean ± SEM, *p* < 0.05 was set as the threshold for significance by one-way ANOVA followed by post hoc comparisons using Tukey’s test for multiple groups’ comparisons, *p < 0.05, **p < 0.01, ***p < 0.001.

## Data Availability

The raw sequencing data generated in this study have been deposited in NCBI Sequence Read Archive (http://www.ncbi.nim.nih.gov/sra) under the accession numbers PRJNA826223.

## Abbreviations

BBB: Blood-brain barrier
DMEM: Dulbecco’s modified Eagle’s medium
FMT: Fecal microbiota transplantation
FBS: Fetal bovine serum
HDAC3: Histone deacetylase3
IOD: Integral optical density
LPS: Lipopolysaccharide
LDA: Linear discriminant analysis
MWM: Morris water maze
Mel: Melatonin
NF-κB: Nuclear factor-κB
PCA: Principal component analysis
PCoA: Principal coordinate analysis
SD: Sleep deprivation
SCFAs: Short-chain fatty acids
TLR4: Toll-like receptor 4
UPGMA: Unweighted pair-group method with arithmetic mean
