# Supplementary material for: Gut microbiota-derived metabolites mediate the neuroprotective effect of melatonin in cognitive impairment induced by sleep deprivation
Source: Microbiome. 2023 Jan 31;11:17. doi: 10.1186/s40168-022-01452-3 (PMC9887785; doi:10.1186/s40168-022-01452-3)
Supplement: Supplementary file 2 — Additional file 1: Supplemental Figure 1. The gut microbiota mediated the neuroprotective effect of melatonin in neuronal apoptosis induced by sleep deprivation. (A-D) Relative protein levels of Bcl-2, Bax and cleaved caspase-3 in the hippocampus (n = 6). CON-FMT: receiving control microbiota FMT mice, SD-FMT: receiving sleep deprivation microbiota FMT mice, SD+Mel-FMT: receiving SD+Mel (20 mg/kg) microbiota FMT mice. The data represent the mean ± SEM, p < 0.05 was set as the threshold for significance by one-way ANOVA followed by post hoc comparisons using Tukey’s test for multiple groups’ comparisons, *p < 0.05, **p < 0.01, ***p < 0.001. Supplemental Figure 2. Composition of the colonic microbiota in FMT-treated mice. (A) OTU number, (B) Simpson index, (C) Shannon index. CON-FMT: receiving control microbiota FMT mice, SD-FMT: receiving sleep deprivation microbiota FMT mice, SD+Mel-FMT: receiving SD+Mel (20 mg/kg) microbiota FMT mice. The data represent the mean ± SEM, p < 0.05 was set as the threshold for significance by one-way ANOVA followed by post hoc comparisons using Tukey’s test for multiple groups’ comparisons, *p < 0.05, **p < 0.01, ***p < 0.001. Supplemental Figure 3. The levels of LPS in the hippocampus (n = 7). CON: control group, SD: sleep deprivation group, SD + Mel: SD + melatonin (20 mg/kg) supplement group. The data represent the mean ± SEM, p < 0.05 was set as the threshold for significance by one-way ANOVA followed by post hoc comparisons using Tukey’s test for multiple groups’ comparisons, *p < 0.05, **p < 0.01, ***p < 0.001. [file 40168_2022_1452_MOESM1_ESM.docx]

**Gut microbiota-derived metabolites mediate the neuroprotective effect of melatonin in cognitive impairment induced by sleep deprivation**

**Supplemental Figure 1**

**
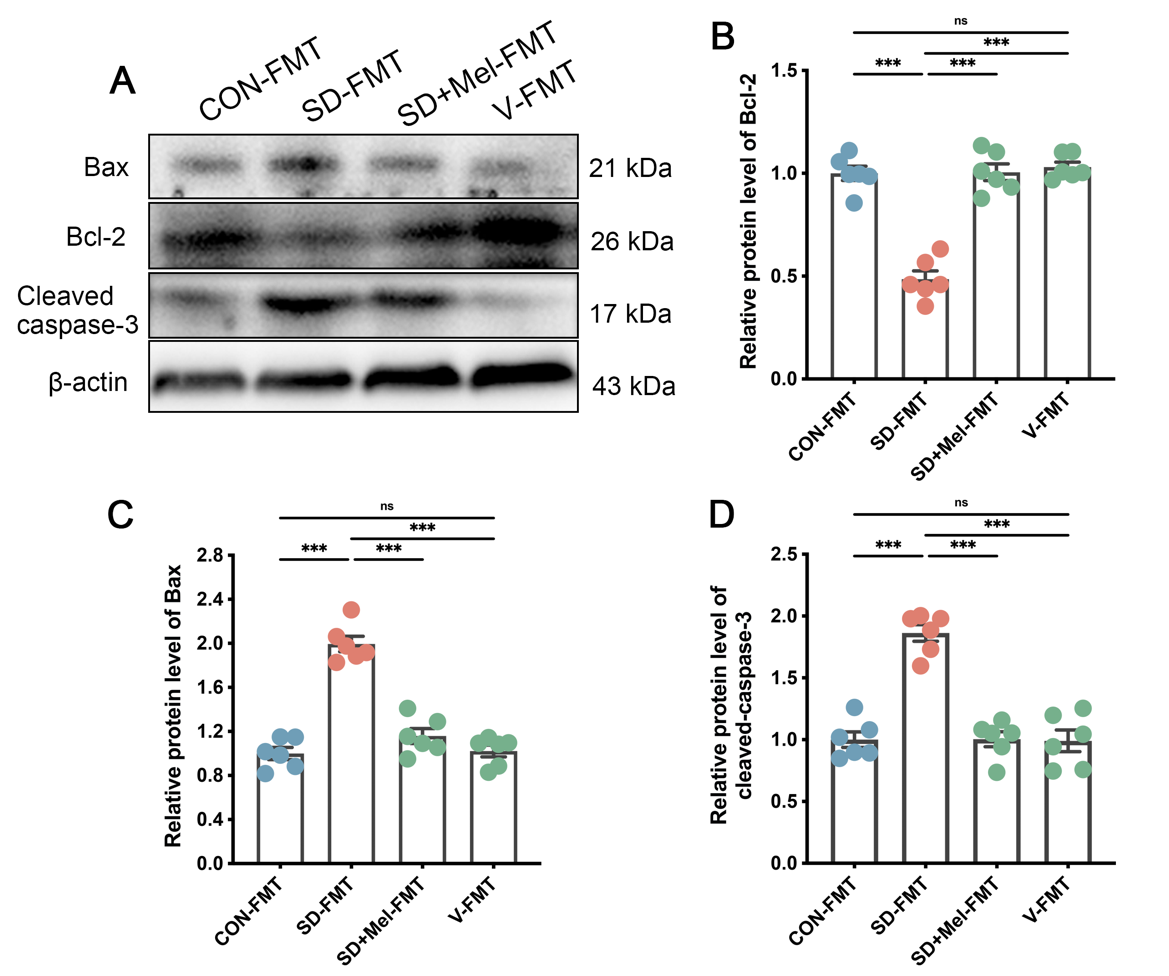
**

**Figure 1 The gut microbiota mediated the neuroprotective effect of melatonin in neuronal apoptosis induced by sleep deprivation.**

(A-D) Relative protein levels of Bcl-2, Bax and cleaved caspase-3 in the hippocampus (n = 6). CON-FMT: receiving control microbiota FMT mice, SD-FMT: receiving sleep deprivation microbiota FMT mice, SD+Mel-FMT: receiving SD+Mel (20 mg/kg) microbiota FMT mice. The data represent the mean ± SEM, p < 0.05 was set as the threshold for significance by one-way ANOVA followed by post hoc comparisons using Tukey’s test for multiple groups’ comparisons, *p < 0.05, **p < 0.01, ***p < 0.001.

**Supplemental Figure 2**


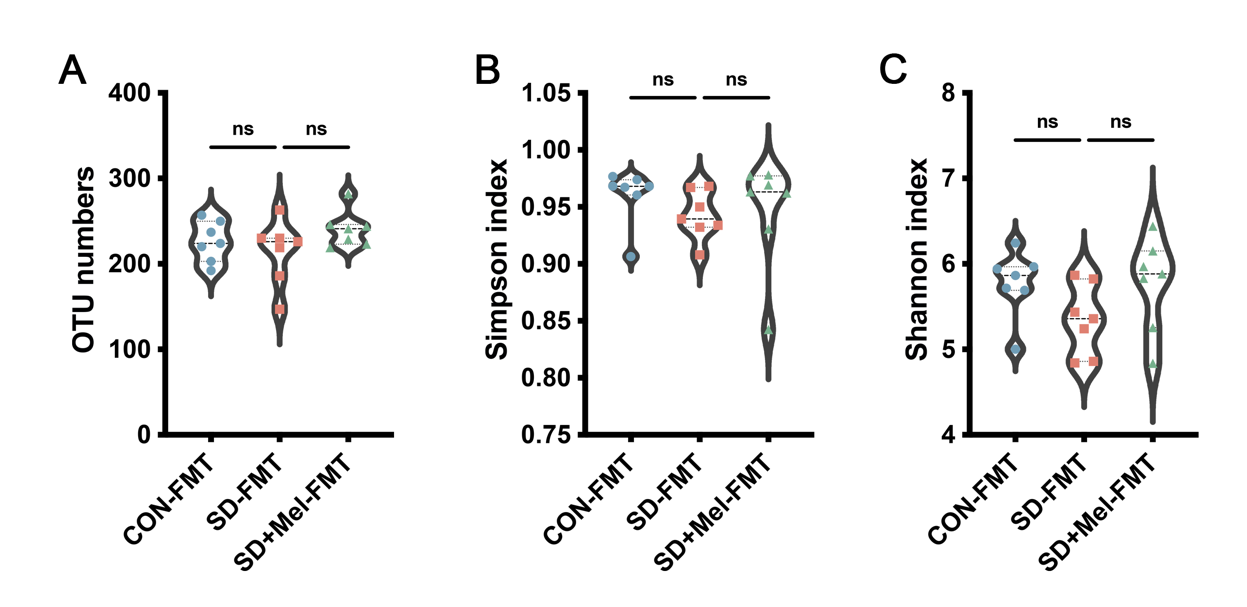


**FIGURE 2 Composition of the colonic microbiota in FMT-treated mice.** (A) OTU number, (B) Simpson index, (C) Shannon index. CON-FMT: receiving control microbiota FMT mice, SD-FMT: receiving sleep deprivation microbiota FMT mice, SD+Mel-FMT: receiving SD+Mel (20 mg/kg) microbiota FMT mice. The data represent the mean ± SEM, p < 0.05 was set as the threshold for significance by one-way ANOVA followed by post hoc comparisons using Tukey’s test for multiple groups’ comparisons, *p < 0.05, **p < 0.01, ***p < 0.001.

**Supplemental Figure 3**


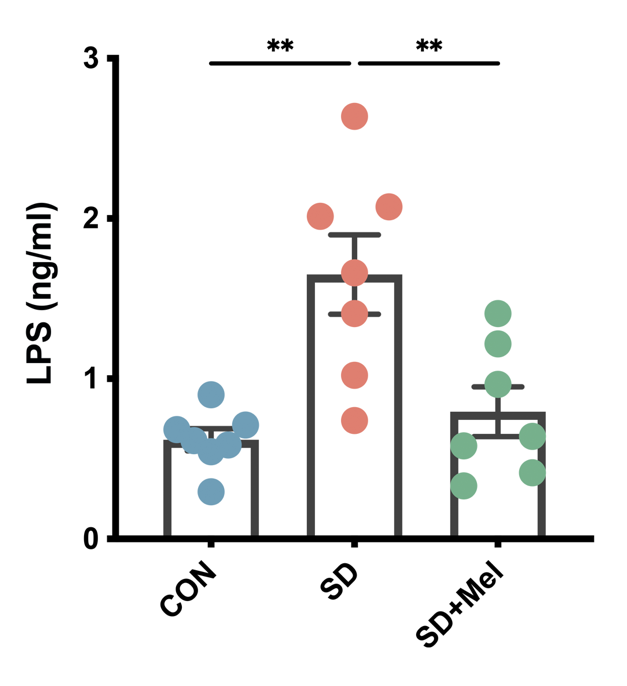


**FIGURE 3** The levels of LPS in the hippocampus (n = 7). CON: control group, SD: sleep deprivation group, SD + Mel: SD + melatonin (20 mg/kg) supplement group. The data represent the mean ± SEM, p < 0.05 was set as the threshold for significance by one-way ANOVA followed by post hoc comparisons using Tukey’s test for multiple groups’ comparisons, *p < 0.05, **p < 0.01, ***p < 0.001.
